# The C-terminus of the cargo receptor Erv14 affects COPII vesicle formation and cargo delivery

**DOI:** 10.1242/jcs.260527

**Published:** 2023-02-06

**Authors:** Daniel Lagunas-Gomez, Carolina Yañez-Dominguez, Guadalupe Zavala-Padilla, Charles Barlowe, Omar Pantoja

**Affiliations:** ^1^ Centro de Investigación en Dinámica Celular, Instituto de Investigación en Ciencias Básicas y Aplicadas, Universidad Autónoma del Estado de Morelos, Av. Universidad 1001, Col. Chamilpa, Cuernavaca, Morelos 62210, Mexico; ^2^ Instituto de Biotecnología, Universidad Nacional Autónoma de México, Av. Universidad 2001, Cuernavaca, Morelos 62210, México; ^3^ Department of Biochemistry, Geisel School of Medicine, Dartmouth College, Hanover, NH 03755-3844, USA

**Keywords:** Erv14, Cornichon, Phosphorylation, COPII vesicles, Membrane trafficking

## Abstract

The endoplasmic reticulum (ER) is the start site of the secretory pathway, where newly synthesized secreted and membrane proteins are packaged into COPII vesicles through direct interaction with the COPII coat or aided by specific cargo receptors. Little is known about how post-translational modification events regulate packaging of cargo into COPII vesicles. The *Saccharomyces cerevisiae* protein Erv14, also known as cornichon, belongs to a conserved family of cargo receptors required for the selection and ER export of transmembrane proteins. In this work, we show the importance of a phosphorylation consensus site (S134) at the C-terminus of Erv14. Mimicking phosphorylation of S134 (S134D) prevents the incorporation of Erv14 into COPII vesicles, delays cell growth, exacerbates growth of *sec* mutants, modifies ER structure and affects localization of several plasma membrane transporters. In contrast, the dephosphorylated mimic (S134A) had less deleterious effects, but still modifies ER structure and slows cell growth. Our results suggest that a possible cycle of phosphorylation and dephosphorylation is important for the correct functioning of Erv14.

## Teaser

**Summary:** COPII vesicle formation and trafficking of plasma membrane proteins depend on the phosphorylated state of S134 in Erv14

## INTRODUCTION

The early secretory pathway in eukaryotic cells is a process important for the delivery of membrane and soluble proteins to various cellular destinations. Once proteins have been correctly folded and modified in the endoplasmic reticulum (ER), they are sorted from ER-resident proteins and selectively incorporated into COPII-coated vesicles that bud from the ER membrane at ER-exit sites (ERES) to be transported to the Golgi (Brandizzi and Barlowe, 2013), although some proteins exit the ER non-selectively via bulk flow (Martínez-Menárguez et al., 1999; Thor et al., 2009). COPII-coated vesicle generation in *Saccharomyces cerevisiae* begins when the GTPase Sar1 is activated by the guanine nucleotide exchange factor (GEF) Sec12, causing its incorporation into ER membranes where it recruits the inner coat layer complex, namely, the Sec24–Sec23 complex. Coat polymerization and vesicle budding occur when Sec23–Sec24 recruits a second complex, the tetramer composed of Sec13–Sec31 that forms the outer coat layer. These events lead to the hydrolysis of GTP on Sar1 and vesicle budding (Sato and Nakano, 2007; Zanetti et al., 2012). The loading of cargo proteins into these vesicles can occur either directly by binding specific COPII subunits, such as Sec24, which binds sorting signals in cargo proteins, or through the participation of proteins called cargo receptors (CRs), which link cargo proteins indirectly to the COPII coat (Dancourt and Barlowe, 2010; Herzig et al., 2012; Miller et al., 2002). Among the cargo receptors that have been characterized are members of the p24 family (Emp24, Erv25, Erp1 and Erp2), which form the p24 complex and selectively recruit glycosylphosphatidylinositol (GPI)-anchored proteins (Muñiz et al., 2000), as well as Erv29 or mammalian ERGIC-53-related family members, which transport soluble secreted glycoproteins (Appenzeller et al., 1999; Belden and Barlowe, 2001). The Erv14 (also known as cornichon) family comprises transmembrane proteins that cycle between the ER and early GA and are involved in the selection of membrane proteins into COPII vesicles (Pagant et al., 2015), most of which are proteins that reside in the plasma membrane (PM) (Herzig et al., 2012). Previous reports on the functioning of *S. cerevisiae* Erv14 identified a cytosolic motif (IFRTL) that is required for COPII binding and ER exit of the receptor (Powers and Barlowe, 2002). Subsequent studies have proposed that cargo selection occurs through recognition of long transmembrane domains (TMDs), which are characteristic of PM proteins (Herzig et al., 2012). It has also been proposed that some cargoes bind to residues in the second TMD of Erv14, which together with a new site identified in Sec24, is proposed to form a dual interaction with both cargo proteins and Erv14. In this view, Erv14 could act as a classical cargo receptor, binding simultaneously to cargo proteins and the COPII coat to drive trafficking (Pagant et al., 2015).

However, little is known about the regulation of the anterograde transport between the ER and the Golgi complex by post-translational modifications, such as protein phosphorylation (Davis et al., 2016; Koreishi et al., 2013; Lord et al., 2011). In *S. cerevisiae*, it has been reported that some COPII coat subunits are phosphorylated by the serine/threonine kinase Hrr25 (a CK1δ ortholog), allowing coat release and vesicle fusion with the Golgi (Lord et al., 2011). On the other hand, it has been reported that Sit4, a serine/threonine phosphatase, is important for COPII coat dephosphorylation. In the *sit4Δ* mutant, the COPII proteins are hyperphosphorylated and their subcellular locations are modified (Bhandari et al., 2013).

In a previous report, we demonstrated the presence and importance of a conserved acidic motif at the C-terminus of plant and yeast Erv14/cornichon homologs, that it is necessary for the binding of the cargo receptor to cargo proteins and their trafficking to the PM (Rosas-Santiago et al., 2017). Additional analysis of the C-terminus of Erv14 suggested the presence of a motif that could function as a consensus sequence (ESXDD) for casein kinase 2 (CK2). In this work, we show that possible phosphorylation and dephosphorylation of S134 is necessary for the correct packaging of Erv14 into the COPII vesicles and as a consequence, the proper trafficking of its cargo proteins to the PM.

## RESULTS

### Possible phosphorylation of Erv14 at the C-terminus

**Fig. 1.**
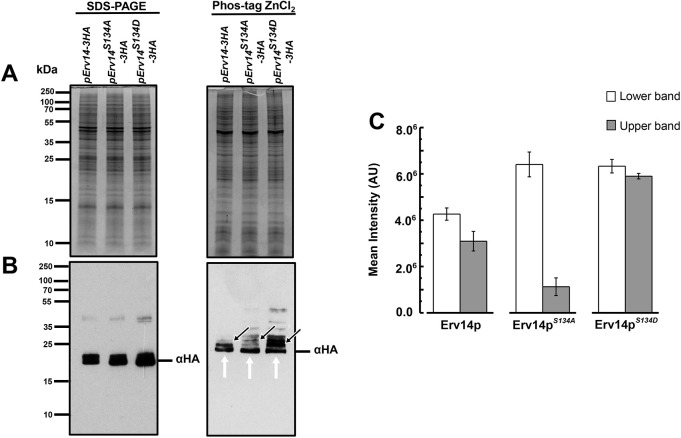
**Erv14 is phosphorylated at the C-terminus.** (A) Microsomal proteins (p13 fraction) from HA-tagged Erv14-, Erv14*^S134A^*- or Erv14*^S134D^*-expressing cells were separated in SDS-PAGE (left) or Zn^2+^-Phos-tag (right) gels. (B) HA-tagged Erv14 proteins were identified an anti-HA antibody. Arrows indicate phosphorylated (black) and non-phosphorylated (white) isoforms, respectively. (C) Bar plot shows the ratio intensity from the top to that of the lower band corresponding to the recognition by anti-HA antibody from HA-tagged Erv14-, Erv14*^S134A^*- and Erv14*^S134D^*-expressing cells. Data are the mean±s.d. from three different preparations.

We recently identified an acidic motif (ESXDD) at the C-terminus of Erv14 that functions as an interaction site with its cargo proteins. This acidic motif is only present in plants and fungi homologs, but not in higher organisms (Rosas-Santiago et al., 2017). To characterize more deeply the role of the C-terminus of Erv14, we identified a putative phosphorylation site at serine 134 (S134) (Fig. S1). S134 is located within the acidic motif which could function as a consensus sequence (ESXDD) for casein kinase 2 (CK2) (St-Denis et al., 2015). This observation opened the possibility that the post-translational modification of Erv14 could play a role in the trafficking of membrane proteins. In order to identify if S134 was phosphorylated, we analyzed the band corresponding to the HA-tagged protein by liquid chromatography tandem mass spectrometry (LC-MS/MS), but were unable to identify any protein modification, but confirmed the presence of Erv14 according to the identification of the peptides (IYNKVQLLDATEIFR) and (VQLLDATEIFR) (Fig. S2). In view of these results, we attempted to identify whether Erv14 was phosphorylated using the ZnCl_2_-Phos-tag SDS-PAGE (Wako Chemicals, USA) technique, which is an electrophoresis approach that allows the separation of phosphorylated and non-phosphorylated proteins using conventional SDS-PAGE procedures (Kinoshita and Kinoshita-Kikuta, 2011). For this, we examined possible changes in the migration patterns of the three Erv14 protein versions. As shown in Fig. 1A, the total protein profile between the cells expressing Erv14, Erv14*^S134A^* or Erv14*^S134D^* mutants, was very similar on SDS-PAGE or the Zn^2+^-Phos-tag gel, but different between the two separation matrixes, indicating the modified migration of many proteins in the latter (Fig. 1A). To identify modifications on the mobility of Erv14, Erv14*^S134A^* or Erv14*^S134D^* on the two separation matrixes, we analyzed their migration by western blotting. In the conventional SDS-PAGE gel, no difference was observed in the banding pattern between the Erv14 and the mutants Erv14*^S134A^* or Erv14*^S134D^* (Fig. 1B). In comparison, we observed in the Zn^2+^-Phos-tag gel the presence of two Erv14 isoforms, with the top one probably corresponding to the phosphorylated form (Fig. 1B). For the Erv14*^S134A^* and Erv14*^S134D^* mutants, these two bands showed different patterns, with a marked decreased in the signal for the top band from Erv14*^S134A^*, whereas a stronger signal was associated with Erv14*^S134D^* (Fig. 1B). Additional bands were revealed by the anti-HA antibody, with an apparent increase in the abundance in Erv14*^S134D^* (Fig. 1B). To confirm these results, the ratio between the intensity of the top to that of the lower band was quantified from the Zn^2+^-Phos-tag gel (Fig. 1C). This analysis showed that the amount of the top isoform in Erv14 was 35% less abundant than the lower band, a condition that clearly changed for the Erv14*^S134A^* mutant, where the top band almost disappeared (18%) (Fig. 1C). For the Erv14*^S134D^* mutant the abundance of both isoforms was very similar (92%; Fig. 1C), suggesting the possible phosphorylation of Erv14 at S134.

### A phospho-mimetic state of S134 affects the trafficking of PM proteins

**Fig. 2.**
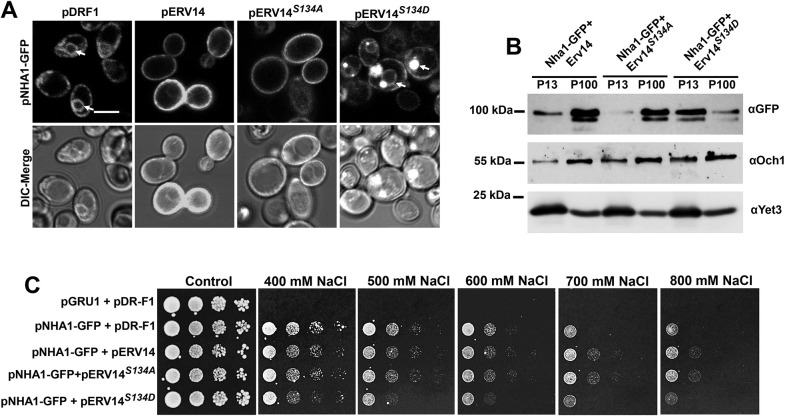
**The phospho-mimic mutation S134D in Erv14 affects the targeting of the Nha1 exchanger to the PM.** (A) PM localization of the antiporter Nha1–GFP was observed in BYT45*erv14Δ* cells transformed with wild type pERV14 or pERV14*^S134A^*, but not with pDR-F1 (empty) or *pERV14^S134D^*. Arrows indicate perinuclear ER. Scale bar: 2 µm. (B) Confirmation of proper localization of Nha1 to the PM by pERV14 or pERV14*^S134A^*, but not by pERV14*^S134D^*. Och1 and Yet3 were used as markers for the Golgi and ER, respectively. (C) Salt sensitivity of the BYT45*erv14Δ* cells was rescued by co-transformation with pNHA1-GFP and pERV14 or pERV14*^S134A^*, but not by co-transformation with pERV14*^S134D^*. Images are representative of at least three experimental repeats.

To obtain additional evidence on the possible phosphorylation of S134, we took an alternative approach by generating mutations in S134. We mimicked the phosphorylated and non-phosphorylated states of S134 with the mutations S134D (Erv14*^S134D^*) and S134A (Erv14*^S134A^*), respectively. When we transformed Erv14*^S134A^* or Erv14*^S134D^* into the strain BY4741*Δerv14* and monitored cell growth rate, we observed a slower growth compared to the wild-type, and even slower for cells transformed with the empty vector pDR-F1 (Fig. S3). These data indicate the importance of S134 for the correct functioning of Erv14. In view of this evidence and previous reports where Erv14 has been identified as an important cargo receptor, mainly for PM proteins (Herzig et al., 2012; Pagant et al., 2015), we tested whether the mutation of S134 modified the targeting of the Na^+^ exchanger Nha1 to the PM. In the salt-sensitive strain BYT45*erv14Δ* (Navarrete et al., 2010; Rosas-Santiago et al., 2016, 2017), co-expression of Nha1–GFP with the wild-type Erv14 or the Erv14*^S134A^* mutant, showed localization of the exchanger to the PM (Fig. 2A); conversely, the antiporter was retained at the ER upon co-expression with the phospho-mimetic mutant Erv14*^S134D^* or the empty plasmid pDR-F1 (Fig. 2A). These results indicate that the phosphorylation of S134 prevented the correct trafficking of Nha1 to the PM. To corroborate these results, we next analyzed the localization of the antiporter at the ER (P13) or Golgi and PM (P100) fractions by differential centrifugation. As observed in Fig. 2B, when *NHA1-GFP* was co-transformed with wild-type *ERV14* or *ERV14^S134A^* into the BYT45*erv14Δ* strain, the antiporter was detected mostly in the PM (P100) fraction, indicative of normal trafficking. In contrast, when Nha1–GFP was co-expressed with the mutant Erv14*^S134D^*, the antiporter remained in the ER (P13) fraction (Fig. 2B). These results were confirmed by quantifying the intensity of the bands that showed a low abundance of Nha1 at the PM fraction relative to the ER in cells co-expressing Nha1–GFP and Erv14*^S134D^* (Fig. S4). This demonstrates that the phosphorylated mimetic of S134 prevents the correct trafficking of its client. Additional confirmation on the importance of the phosphorylation state of Erv14 for the correct targeting of Nha1 to the PM was derived from assessing the sensitivity of the yeast cells to Na^+^. Co-expression of Nha1–GFP and Erv14 or the Erv14*^S134A^* mutant, rescued the tolerance of BYT45*erv14Δ* cells to Na^+^, in contrast to what was seen with cells co-transformed with *NHA-GFP* and *ERV14^S134D^*, which were more sensitive to the cation (Fig. 2C). Together, these results are indicative of an impaired delivery of Nha1 to the PM by the Erv14*^S134D^* mutant.

**Fig. 3.**
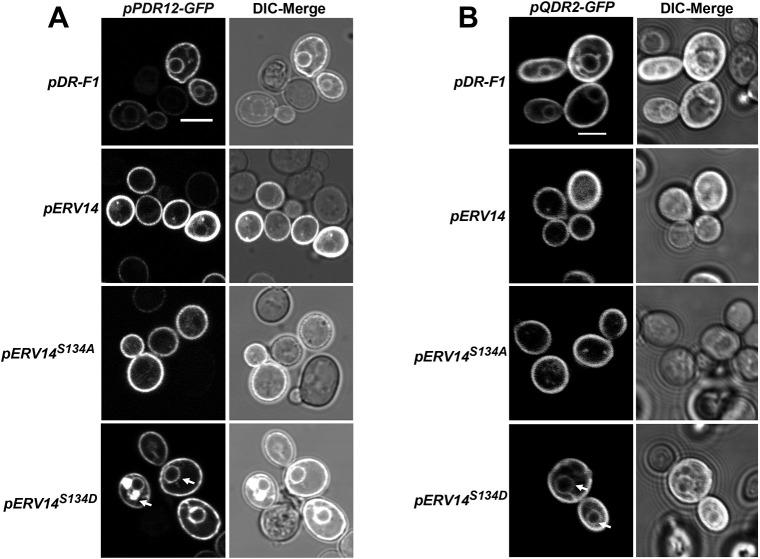
**The S134D mutation at the C-terminus in Erv14 prevents export of PM cargoes.** Trafficking to the PM of the ABC transporter Pdr12-GFP or the MFS protein Qdr2-GFP in the BY4741*pdr12Δerv14Δ* (A) or BY4741*qdr2Δerv14Δ* (B) cells only occurred in cells co-transformed with wild type pERV14 or pERV14*^S134A^*, but not with the empty vector (pDR-F1) or pERV14*^S134D^*, where cargo proteins showed a perinuclear ER retention (arrows). Scale bars: 2 µm. Images are representative of at least three experimental repeats.

Erv14 has been identified as a cargo receptor that is required for efficient ER export of several proteins, the vast majority of which locate to the PM (Herzig et al., 2012; Pagant et al., 2015; Rosas-Santiago et al., 2017). To analyze if more clients whose transport out of the ER is dependent on Erv14 were affected by the phosphorylation state of the receptor, we examined Pdr12, a weak acid-efflux ATPase from the ABC family (Piper et al., 1998) and Qdr2, a drug/proton antiporter of the MFS family (Vargas et al., 2004). The co-transformation of BY4741*pdr12Δerv14Δ* cells with *PDR12-GFP* and *ERV14* or *ERV14^S134A^* did not affect the delivery of the Pdr12–GFP to the PM (Fig. 3A). In comparison, co-expression of Pdr12–GFP with Erv14*^S134D^* or the empty pDR-F1 vector caused retention of the ABC transporter at the ER, as clearly shown by the fluorescence signal around the nucleus and the cell periphery, corresponding to the perinuclear and cortical localization of the ER in yeast cells (Fig. 3A). Similar results were obtained with Qdr2, where we observed correct targeting of the exchanger to the PM with either Erv14 or Erv14*^S134A^* (Fig. 3B), but not with Erv14*^S134D^* or the empty vector (Fig. 3B). These results confirmed that the phospho-mimetic state of Erv14 controls the traffic of its cargo proteins.

### S134 is not required for interaction with the cargo proteins

**Fig. 4.**
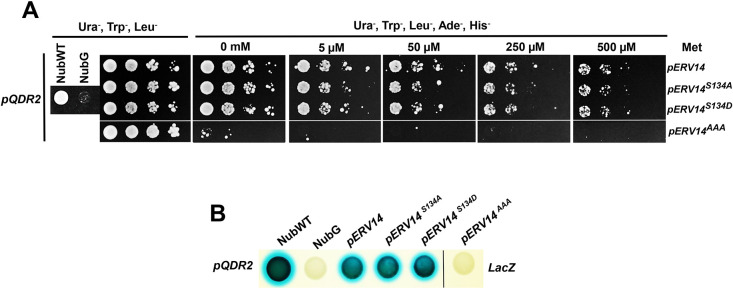
**Mutations in S134 do not affect binding with cargo proteins.** (A) Interaction between Qdr2 and Erv14 isoforms was not affected by the mutation of S134 and showed similar strength at increasing concentrations of methionine. (B) Corroboration of the interactions was demonstrated by activation of LacZ and revealed with X-Gal as substrate (LacZ). NubWT and NubG were used as false negative and false positive controls, respectively. Data are representative of at least three repetitions.

So far, our results demonstrate that the phospho-mimic form impaired delivery of cargo proteins to the PM. We next reasoned that the phosphorylation state of S134 in Erv14 could control or function as a cargo-binding site. We sought to test this hypothesis by monitoring Erv14–cargo interaction using the membrane-based split-ubiquitin yeast two-hybrid system (mbSUS) (Stagljar et al., 1998). For this, we employed Qdr2 as bait (Cub clone), which we had previously identified as interacting with Erv14 (Rosas-Santiago et al., 2017), and the point mutants at S134 as prey (Nub clones). Taking advantage of the repressible Met promoter present in the Cub constructs, we titrated the strength of the interaction employing increasing concentrations of Met (Grefen et al., 2009). Erv14 and Qdr2 showed robust interaction, as indicated by the growth of diploid cells in the selection medium (Ura**^−^**, Trp**^−^**, Leu**^−^**, Ade**^−^** and His**^−^**) (Fig. 4A). The growth of diploid cells was not affected by either of the two mutations Erv14*^S134A^* or Erv14*^S134D^*, indicating that modifications at S134 did not influence the interaction between receptor and cargo. Moreover, the strength of these interactions was similar, as indicated by the similar inhibition of cell growth caused by the increasing concentrations of methionine (Fig. 4A). To confirm the interaction of Qdr2 and the Erv14 isoforms, we tested the activation of the *lacZ* gene by incubating the cells in presence of X-gal. As observed in Fig. 4B, the development of a blue signal in diploid cells expressing Qdr2 and wild-type Erv14 or mutants forms, was of similar intensity, suggesting a similar interaction between the three Erv14 isoforms and the Qdr2 cargo. As a control, we assayed the interaction between Erv14 mutated in the three acid amino acids (E133, D136 and D137; Erv14*^AAA^*) and Qdr2 observing the lack of interaction between the two proteins (Fig. 4A), confirming the importance of these residues as part of the domain responsible for the protein interaction of the receptor with its cargo, as reported previously (Rosas-Santiago et al., 2017), and demonstrating that the results with the S134 mutants were specific. Together, these results suggest that S134 does not play an important role in the interaction between the Erv14 cargo receptor and its cargoes.

### Mutation of S134 modifies the subcellular location of Erv14 and induces structural changes in the ER

**Fig. 5.**
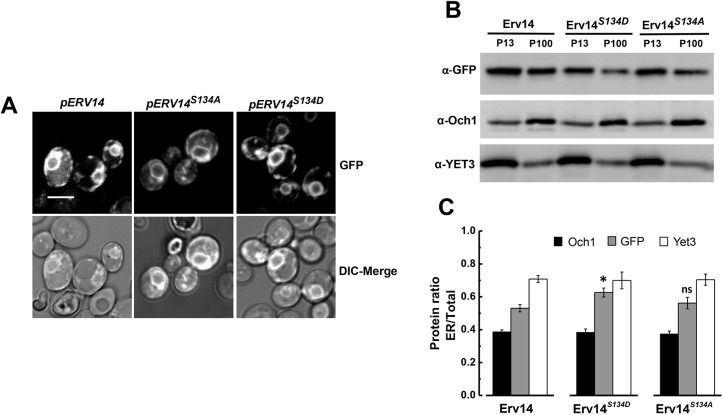
**The S134D mutation modified the subcellular location of Erv14.** (A) GFP-tagged Erv14 isoforms were expressed in the BY4741*erv14Δ* strain. ER and Golgi membrane localization of GFP-tagged Erv14, Erv14*^S134A^* or Erv14*^S134D^* showed similar subcellular localization. Scale bar: 2 µm. Images are representative of at least three experimental repeats. (B) Erv14 membrane distribution in ER or Golgi fractions from GFP-tagged wild-type and Erv14*^S134A^* isoforms was similar, observing a decrease of Erv14*^S134D^* at the Golgi fraction*.* Yet3 and Och1 were used as markers for the ER and Golgi, respectively. (C) Bar plot showing the ratio of each protein at the ER against the total (ER+Golgi). Data are the mean±s.d. from three different preparations. ns, *P*>0.05, *P<0.05, compared to wild type (unpaired two-tailed Student's *t*-test).

**Fig. 6.**
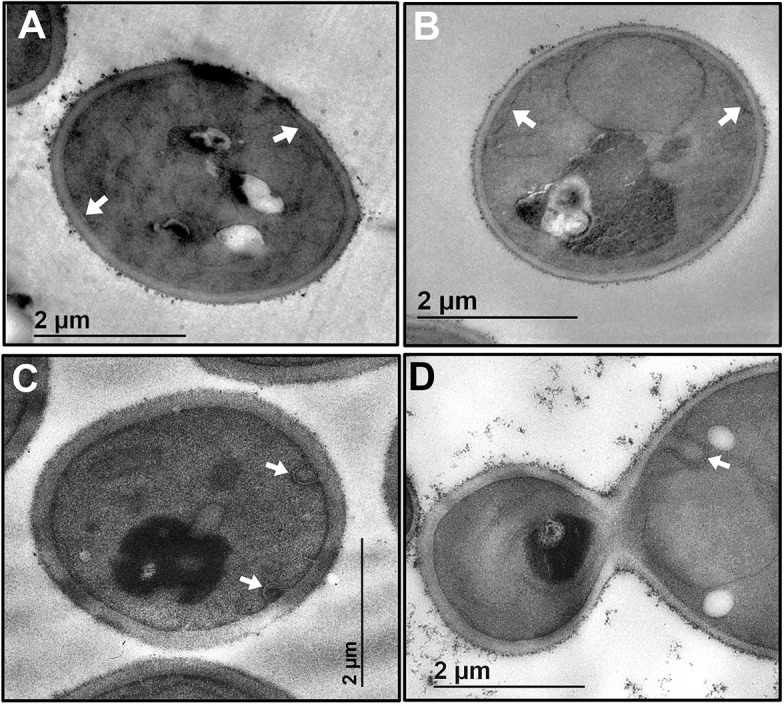
**Mutations at S134 induced modifications in ER structure.** TEM micrographs from BY4741*erv14Δ* cells expressing Erv14 or the mutants Erv14^S134A^ or Erv14*^S134D^*. (A) In Erv14-expressing cells, the cortical ER was observed as a continuous thread closely attached to the PM (arrows). (B) In mutant *erv14Δ* cells, the cortical ER separated from the PM. In cells expressing the mutants Erv14*^S134A^* (C) or Erv14*^S134D^* (D), cortical ER showed deformation into omega-like structures (arrows). Images are representative of at least three experimental repeats.

Erv14 is a transmembrane protein that localizes to the ER and Golgi compartments and is proposed to cycle between these two organelles through the COPII and COPI systems (Powers and Barlowe, 1998, 2002). In view of our previous results, we wondered whether mistargeting of Erv14 cargoes could be explained by alterations in localization of the receptor. To analyze this possibility, we generated GFP fusions at the C-terminus of Erv14 and the S134 mutants to follow their localization *in vivo* by confocal microscopy. Erv14–GFP was found mainly in both the cortical and the perinuclear ER, with some dots that could be associated with the Golgi, as previously observed (Rosas-Santiago et al., 2017) (Fig. 5A). The subcellular localization of the mutants Erv14*^S134A^* or Erv14*^S134D^* seemed to be like that observed for the wild-type receptor (Fig. 5A). Upon monitoring trafficking of Erv14 between the ER and Golgi membranes of the wild-type Erv14 and S134 mutants by membrane fractionation and western blotting, we found that abundance of wild-type Erv14 was similar in the ER and the Golgi fractions, indicative of normal trafficking (Fig. 5B). By contrast, localization of the phospho-mimetic Erv14*^S134D^* mutant was shifted to the ER fraction (P13), indicating its retention at the ER (Fig. 5B). For the Erv14*^S134A^* mutant, no clear change in membrane distribution was observed (Fig. 5B). The unchanged distribution of the Golgi resident Och1 and the ER marker Yet3 (Fig. 5B), confirmed that the changes observed for the Erv14*^S134D^* mutant were specific. Additional confirmation for these results was obtained by quantifying the band intensities from the Western blots. For this, the ratios for each protein in the ER (P13 fraction) with respect to the total protein (ER plus Golgi; P13 and P100) were quantified, showing that the ratio for the mutant Erv14*^S134D^* was 20% higher than that for Erv14 (Fig. 5C). For the S134A mutant, a smaller ratio was observed, but it was not significant (Fig. 5C). Based on these results*,* we explored possible changes caused by the mutations of Erv14 at the ultrastructural level. The wild-type (Erv14) cells showed a clear continuous cortical ER at the TEM level (Fig. 6A). In comparison, cells expressing Erv14*^S134A^* or Erv14*^S134D^* showed a deformed cortical ER, with omega-like structures distributed along this organelle (Fig. 6C,D). These defects were not observed in the mutant strain *erv14Δ*, which showed a separation of the cortical ER from the cell's periphery (Fig. 6B). Data analysis from several cells from each strain clearly showed that the ER deformations were associated with the Erv14*^S134A^* or Erv14*^S134D^* point mutants, but not with the null *erv14Δ* mutant (Fig. S5). These structural changes at the ER membrane caused by mutation of S134 could be due to modifications in trafficking between ER and Golgi, as has been observed for early *sec* mutants that impair vesicle formation (Kaiser and Schekman, 1990; Miller et al., 2002).

### The phospho-mimetic form of Erv14 impairs the formation of COPII vesicles

**Fig. 7.**
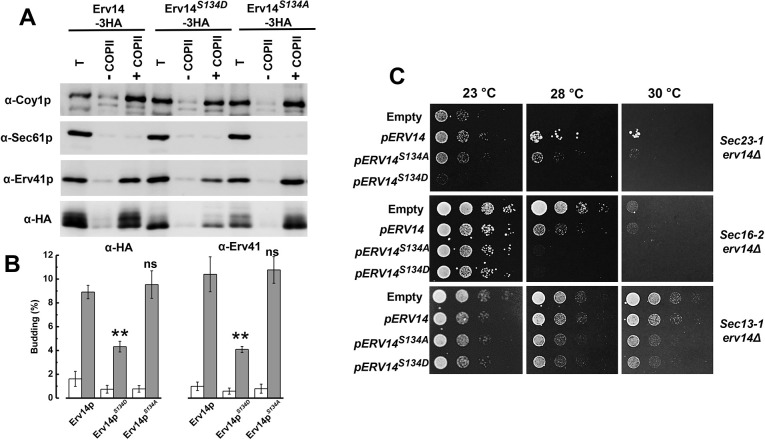
**Mutations in S134 affected packaging of Erv14 into COPII vesicles and prevented the rescue of thermosensitive *sec erv14* mutants by *ERV14*.** (A) Semi-intact BY4742*erv14Δ* cells expressing the wild-type cargo receptor (Erv14–HA), or the mutants Erv14*^S134A^–*HA or Erv14*^S134D^*–HA were incubated with (+) or without (−) purified COPII proteins to monitor vesicle budding. One tenth of the total budding reaction (T) was included as a control. Budded vesicles were isolated and resolved on a 14% polyacrylamide gel and immunoblotted with antibodies specific for Erv41, Coy1 (positive control), Sec61 (negative control) and HA. (B) Quantification of COPII packaging efficiency from three essays as in A without (open bars) or in the presence of COPII proteins (solid bars). Vesicle packaging was evaluated with anti-HA antibody for Erv14 (left) or anti-Erv41 antibody (right). ns, *P*>0.05, **P<0.001 compared with wild type (unpaired two-tailed Student's *t*-test). (C) Cell growth in thermo-sensitive *sec23-1, sec16-2* and *sec13-1* cells that was rescued by *pERV14* did not occur with the Erv14*^S134A^* or Erv14*^S134D^* mutants. Cells were grown at permissive temperature (23°C), diluted and plated, and grown at either permissive (23°C) or restrictive temperatures (28–30°C). The transformants were grown for 3 days. Representative results from three independent assays.

Export of secretory proteins from the ER is mediated by COPII complexes on the ER membrane, driving the formation and budding of COPII-coated vesicles (Barlowe et al., 1994). Erv14 was identified as being enriched in COPII vesicles and necessary for the incorporation of cargo proteins into COPII vesicles (Pagant et al., 2015; Powers and Barlowe, 1998, 2002). Therefore, we asked whether packaging of Erv14 into COPII vesicles was influenced by mutation of the S134 residue using an *in vitro* assay that recapitulates COPII-dependent vesicle formation (Barlowe et al., 1994). For this, we employed washed semi-intact BY4742*erv14Δ* cells (SICs) expressing HA-tagged Erv14 or the point mutants Erv14*^S134A^* or Erv14*^S134D^*, incubated with or without purified COPII proteins (Sar1, Sec23–Sec24 and Sec13–Sec31), GTP and an ATP regeneration system to drive vesicle budding (Baker et al., 1988; Barlowe et al., 1994). The vesicle fraction was separated from bulk membranes by centrifugation, and 10% of the total membrane input (T) was compared with the sample of the vesicular fraction by immunoblotting. In SICs expressing Erv14 or Erv14*^S134A^*, Erv14 was efficiently packaged into the COPII vesicles, as indicated by the strong signal developed by the anti-HA antibody (Fig. 7A). However, for vesicles isolated from the SICs expressing Erv14*^S134D^*, packing of Erv14 into COPII vesicles was less efficient, according to the weaker anti-HA signal (Fig. 7A). In all three cases, Erv14 packing did not occur in the absence of COPII proteins (Fig. 7A). As a negative control, we used Sec61, an integral membrane protein that resides in the ER and is a subunit of the translocon, which was not detected in the vesicle fraction (Fig. 7A). In contrast, Coy1, a well-characterized membrane protein, was correctly sorted into COPII vesicles (Fig. 7A), which also served as positive control (Anderson et al., 2017; Otte et al., 2001). Unexpectedly, a weaker signal was observed for Erv41 (Fig. 7A). These results were confirmed by quantifying budding efficiency as described in the Materials and Methods (Fig. 7B). ER-derived vesicles incorporated Erv14 or Erv14*^S134A^* at 9% and 10%, respectively (Fig. 7B), which is comparable to results previously reported for Erv14 (Powers and Barlowe, 1998). Conversely, Erv14*^S134D^* was packed at lower level (4%) (Fig. 7B). On the other hand, when the budding for Erv41 was quantified in membranes expressing Erv14 or Erv14*^S134A^* we obtained a level of 10% and 11% efficiency, respectively (Fig. 7B). For the membranes expressing Erv14*^S134D^*, packing of Erv41 was less efficient, showing a level of 4% (Fig. 7B). These values are similar to those reported for the *erv14Δ* deletion strain, where the transport efficiency of gpαF was also reduced when compared to what was seen in the wild type (Powers and Barlowe, 2002). Together, these results indicate that the phospho-mimetic mutation at the C-terminus of Erv14 decreases formation of COPII vesicles.

### Mutation of S134 Erv14 exacerbates the growth of secretory yeast mutants

In a previous report, it was shown that deletion of *ERV14*, when combined with temperature-sensitive mutations involved in COPII vesicle formation, such as mutations in *SEC23*, *SEC16* and *SEC31*, increased the thermosensitivity of the cells, which indicated that Erv14 was involved in the formation of the COPII vesicles (Powers and Barlowe, 2002). To support the results of *in vitro* vesicle budding, we examined whether *ERV14* mutation modified the viability of the temperature-sensitive mutants in the early secretory pathway. We placed *ERV14* and the S134 mutants under the control of the constitutive GADP promoter and introduced each construction into the double mutants *sec23-1 erv14Δ*, *sec13-1 erv14Δ* and *sec16-2 erv14*Δ and grew them at increasing temperatures of 23°C, 28°C and 30°C. As shown in Fig. 7C, the *sec23-1 erv14Δ* strain transformed with *ERV14^S134D^* did not grow at the semi-restrictive temperature of 28°C nor at 30°C, like the cells transformed with the empty vector. In contrast, *sec23-1 erv14Δ* cells showed a clear growth at 23°C when transformed with *ERV14^S134A^*, although not as well as cells transformed with *ERV14* (Fig. 7C). For the *sec16-2 erv14Δ* strain, transformation with *ERV14* reduced cell growth at the non-permissive temperatures of 28°C and 30°C, a response that was exacerbated when the cells were transformed with *ERV14^S134A^* or *ERV14^S134D^* (Fig. 7C). With the *sec13-1 erv14Δ* double mutant, we observed a lower growth of the cells transformed with *ERV14^S134A^* or *ERV14^S134D^*; conversely, when transformed with *ERV14*, cells grew better and similar to those transformed with the empty plasmid (Fig. 7C). Thus, these genetic interactions show that the cells expressing the *ERV14^S134D^* mutant, in particular, inhibited cell growth and corroborated our *in vitro* budding results, which indicate that the S134D mutation in Erv14 influences COPII vesicle formation.

## DISCUSSION

Traffic control of membrane proteins from their place of synthesis, the ER, to their place of residence, is tightly regulated by proteins called cargo receptors. The Erv14 family of proteins has been identified as cargo receptors that facilitate the packaging of membrane proteins into COPII-coated vesicles for trafficking out of the ER (Herzig et al., 2012; Pagant et al., 2015; Powers and Barlowe, 1998, 2002). However, little is known about the regulation of these cargo receptors by post-translational modifications. Recently, we showed that the (**E**SG**DD**) acidic motif at the C-terminus of Erv14 is required for interaction and correct targeting of its cargo proteins (Rosas-Santiago et al., 2017). After further analysis, we identified that the C-terminus possesses a consensus sequence for the activity of casein kinase II (CK2) for phosphorylation of S134 according to the Netphos3.1 motif prediction program (Fig. S1). This domain agrees with empirical data that demonstrated a required presence of an acidic residue at the position located three residues to the C-terminus of the phosphate acceptor site, together with the enhancing effect generated by the presence of an acidic residue on the amino side of the Ser residue, which is preferred by CK2 rather than threonine (Meggio and Pinna, 2003; Salvi et al., 2009; St-Denis et al., 2015). This evidence opened the possibility that the phosphorylation and dephosphorylation of S134 could modify the functioning of Erv14.

By employing mutant analogs for the phosphorylated or dephosphorylated residue S134, we demonstrate that functioning of the Erv14 cargo receptor is affected differentially by both modifications. Although Erv14 membrane localization, ER ultrastructure and interaction with the cargoes were similarly modified by both mutations, only the phospho-mimic S134D mutation significantly reduced the delivery of cargo proteins to the PM that was associated with subtle changes in the distribution of the receptor between the ER and the Golgi. These results suggest that the mutation S134D alters the trafficking of its clients through a mechanism that does not seem to involve a direct protein–protein interaction. This observation contrasts with the direct prevention of the interaction of the receptor with its cargoes that occurred when the acidic amino acids that form part of the phosphorylation domain were mutated (Rosas-Santiago et al., 2017).

Our experiments from *in vitro* assays that reproduced COPII budding, reinforced the view that the mutant Erv14*^S134D^* affects the packaging of Erv14 into COPII vesicles and decreases their formation, which could explain the mistargeting of cargo proteins (Fig. 7). The reduced packaging of Erv41 and Coy1, a cargo receptor and a Golgi-located protein, respectively, that are known to travel or use the COPII system (Anderson et al., 2017; Otte et al., 2001), confirms that the phospho-mimetic mutation in S134 affects the formation of the COPII vesicles (Fig. 7). Supporting this observation are the genetic experiments showing that the expression of Erv14*^S134D^* exacerbated cell growth of temperature sensitive mutants directly involved in COPII formation (*SEC23*, *SEC16* and *SEC13*) (Fig. 7C). Particularly, the strong inhibition of cell growth of the Sec23 mutant can be used to propose that because Erv14 directly interacts with the Sec23–Sec24 complex (Pagant et al., 2015; Powers and Barlowe, 2002), it might be possible that mimicking phosphorylation of S134 at the C-terminus, inhibits or alters binding of Erv14 to COPII subunits, thus interrupting cargo trafficking.

Based on the cornichon molecular model (Nakagawa, 2019) and the one we derived for Erv14 using the AlphaFold algorithm (https://www.deepmind.com; Fig. S6), we can confirm that the C-terminus faces the ER lumen, and the IFRTL domain, which has been shown to be important for the interaction with the Sec23–Sec24 complex (Pagant et al., 2015; Powers and Barlowe, 2002), faces the cytoplasm. It is possible that by simulating phosphorylation of S134 a conformational change in Erv14 is induced, precluding its correct interaction with the Sec23–Sec24 complex, and therefore, the formation of COPII vesicles.

According to the changes observed in the TEM images and the genetic interactions with the *sec* mutants, both Erv14*^S134D^* and Erv14*^S134A^* cause deformations in the ER and worsen cell growth (Fig 6; Fig. S3), suggesting an alteration in the ER-to-Golgi transport, as has been observed for several *sec* mutants (Kaiser and Schekman, 1990; Miller et al., 2002).

Indications that S134 is phosphorylated were obtained by analysis of the changes on mobility of the modified protein employing the Zn^+2^-Phos-tag technique, where the presence of a second band with a retarded movement was observed for Erv14 (Fig. 1). This observation was confirmed by a decrease in abundance of the slower band in the S134A mutant, while observing an increase in the S134D mutant (Fig. 1). Presence of the upper band in the S134D mutant confirms that the mutation seems to be a good representation of the phosphorylated state of Erv14, evidence that helps us to conclude that phosphorylation and dephosphorylation of Erv14 is an important mechanism in the functioning of COPII in the transport of PM proteins. The intensity of the bands associated with Erv14 indicate that the abundance of the phosphorylated protein seems to be slightly lower at the ER, which together with the results from the S134A and S134D mutants, indicate that an alteration in this proportion can have severe consequences in the functioning of the cargo receptor and accordingly, in the formation of COPII vesicles. The detection of additional bands with the S134D mutant might be due to a strong interaction between the carboxylate group from aspartate, and the Zn^+2^-Phos-tag complex. Additional evidence for the modification of Erv14 was obtained by mutating the acidic amino acids (E133, D136 and D137) to disrupt the phosphorylation domain without modifying S134. This modification led to the disappearance of the upper band (Fig. S7), just like the mutation S134A, thus confirming its association with the phosphorylation of Erv14. We attempted to confirm these results by analyzing possible changes in the Erv14 isoforms in the absence of CK2, but, unfortunately, that did not show a clear disappearance of the phosphorylated Erv14 isoform, although a clear decrease in the abundance of the receptor protein was observed in the *cka1Δ* mutant (Fig. S8). These results suggest that an alternative kinase may be responsible for the phosphorylation of Erv14.

**Fig. 8.**
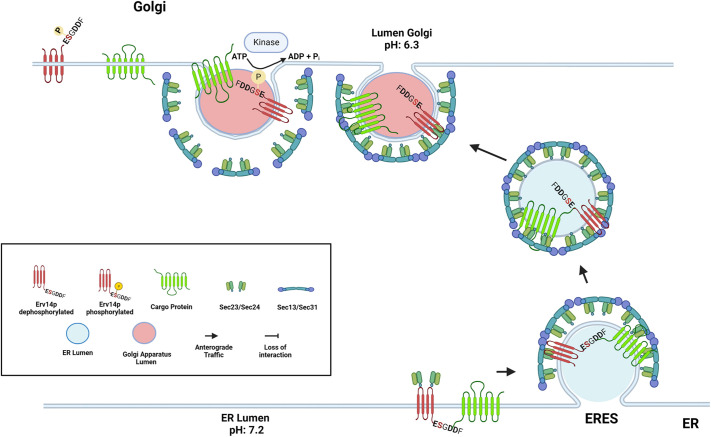
**A working model for the role of phosphorylation of S134 at the C-terminus of Erv14.** At the ER (bottom), Erv14 is found unphosphorylated allowing its binding to Sec24 and its cargoes to be packaged into COPII vesicles. After budding from the ER (middle), the COPII vesicle fuses with the Golgi membrane, where the more acidic luminal pH causes the protonation of the acidic amino acids (**E**SG**DD**) and the release of cargo protein (top-right). Exposure of Erv14 C-terminus to the Golgi lumen will permit phosphorylation of S134 by a Golgi-resident kinase, causing a conformational change in Erv14, allowing its dissociation from the COPII coat and ready for the subsequent retrieval of Erv14 to the ER by COPI vesicles. Figure created with BioRender.com.

Our results raise the question of where the phosphorylation of Erv14 occurs. It has been reported that numerous proteins are phosphorylated by unidentified protein kinases present within the lumen of the Golgi, enzymes that are poorly characterized and have been termed secreted protein kinases (Park et al., 2019). Moreover, both Sec23 and Sec24 are phosphorylated by the Hrr25 kinase, modifications that are proposed to help in the uncoating of COPII vesicles (Bhandari et al., 2013; Lord et al., 2011). This evidence can be used to propose that Erv14 is phosphorylated at the Golgi where it could help in controlling the directionality of Erv14 trafficking into COPI or COPII vesicles for the correct transport of its cargo proteins. We suggest that S134 acts in concert with the acidic amino acids (**E**SG**DD**) at the C-terminus for the controlled transport of cargoes. Owing to the neutral pH of the ER lumen, the acidic amino acids might establish an electrostatic interaction with cargo proteins, allowing their loading into the COPII vesicles, where S134 would not play any role as it would be unphosphorylated. Upon arrival at the Golgi, as it has been previously described (Lord et al., 2011), phosphorylation of several COPII components would occur for the uncoating of the vesicles and their fusion with the acceptor Golgi membrane, exposing the vesicle lumen to a more acidic environment that would release the cargo, expose the phosphorylation domain of Erv14 to be modified by a Golgi resident kinase, to prevent its interaction with the COPII subunits and give directionality to the process (Fig. 8).

## MATERIALS AND METHODS

### Yeast strains and growth media

The yeast strains are listed in Table S1. Yeast cell cultures were grown at 30°C in standard rich medium [YPD; 1% yeast extract (Difco), 2% pectone (Difco) and 2% glucose (Sigma-Aldrich, Carlsbad, CA, USA)] or selective medium YNB containing 0.67% yeast nitrogen base without amino acids (Difco) and 2% glucose, supplemented with amino acids appropriated for auxotrophic growth. Amino acids were used at a concentration of 20 µg/ml (Sigma-Aldrich, Carlsbad, CA, USA). For the mbSUS assay, the synthetic complete (SC) medium was prepared as described previously (Lalonde et al., 2010). The *S. cerevisiae BYT45erv14Δ* mutant strain, which is highly sensitive to alkali metal cations because it has deletion of the cation exporters ENA1–ENA5 and NHA1 was derived from BY4741 cells (Navarrete et al., 2010). The cargo receptor *erv14Δ* mutant and double mutants BY4741*pdr12Δerv14Δ* and BY4741*qdr2Δerv14Δ* were generated previously (Rosas-Santiago et al., 2017). For testing sensitivity to Na^+^, 10-fold serial dilutions of cultures were applied to YNB plates supplemented with NaCl concentration from 0.4 M to 0.8 M; cell growth was recorded for 5 days. For complementation assays of the thermosensitive mutants *Sec23-1 erv14Δ*, *Sec16-2 erv14Δ* and *Sec13-1 erv14Δ*, serial dilutions of cultures were applied with a replica plater (Sigma-Aldrich, R2383) to YNB plates and growth at 23°C, 28°C or 30°C, and cell growth was recorded for 3 days. To monitor growth rate, yeast cells were preinoculated in selective medium and the next day an optical density at 600 nm (OD_600_) of 0.01 was inoculated in YNB medium and growth was monitored every 2 h in a spectrophotometer (Cary 60 UV-Vis, Agilent). Yeast cells were transformed using a standard lithium acetate transformation protocol (Gietz and Woods, 2006).

### Plasmid construction

To generate the point mutations S134A and S134D, the ORF of *ScERV14* was mutated by PCR using Phusion High-Fidelity DNA Polymerase (Thermo Fisher Scientific, Waltham, MA, USA) and employing the specific primers described in Table S2. The PCR products for *ERV14^S134A^* and *ERV14^S134D^* were cloned into the pDONOR207 vector of the Gateway system following manufacturer's instructions (Invitrogen, Carlsbad, CA, USA). The entry clones thus generated were confirmed by restriction analysis with EcoRI and BgllI (Thermo Fisher Scientific, Waltham, MA, USA) and by sequencing. The LR clonase reaction (Invitrogen, Carlsbad, CA, USA) was used to transfer the genes *ERV14, ERV14^S134A^ and ERV14^S134D^* into the multicopy plasmid pAG425-3HA (Addgene plasmid #14250), to generate the HA-tagged fusions pERV14-3HA*,* pERV14^S134A^-3HA and pERV14^S134D^-3HA*.* To complement *ERV14* function in the BYT45*erv14Δ*, BY4741*pdr12Δerv14Δ* and BY4741*qdr2Δerv14Δ* mutant strains, the genes *ERV14*, *ERV14^S134A^* and *ERV14^S134D^* were cloned into the pDR-F1_GW (Addgene plasmid #36026) by an LR clonase reaction (Invitrogen, Carlsbad, CA, USA) generating the constructs pDR-F1-ERV14, pDR-F1-ERV14^S134A^ and pDR-F1-ERV14^S134D^. For the mbSUS assay constructs, the genes *ERV14 ERV14^S134A^* and *ERV14^S134D^* were transferred into the pXN32_GW (Nub clones) vector by an LR clonase reaction (Invitrogene, Carlsbad, CA, USA), generating the pNub-ERV14, pNub-ERV14*^S134A^* and pNub-ERV14*^S134D^*. The pQDR2-Cub (Cub clone) construction used in this work was obtained from a previous work (Rosas-Santiago et al., 2017). To generated GFP-tagged mutant Erv14 proteins, the *ERV14* ORF was amplifying by PCR employing the primers described in Table S2, and the PCR products were cloned into the pGRU1 (Dr Bertrand Daignan-Fornier, Université de Bordeaux, France) by homologous recombination in *S. cerevisiae* BW31a, leaving the *ERV14* ORF under the NHA1 promoter and in-frame with GFP, to yield the constructions pERV14*^S134A^*-GFP and pERV14*^S134D^*-GFP. The constructions were confirmed by restriction analysis with PstI (Thermo Fisher Scientific) and by sequencing, using the oligonucleotides described in the Table S2. The pGRU1-originating plasmid pNHA-985-GFP, pDR12-GFP, pQDR2-GFP and pERV14-GFP used in this work were described previously (Rosas-Santiago et al., 2017).

### Mating based split ubiquitin system

For testing the binding of Erv14 mutants to their cargo proteins, the mbSUS assay was used (Lalonde et al., 2010). The THY.AP4 (*MATa ura3*, *leu2*, lexA::LacZ::*trp1 lexA::HIS3 lexA::ADE2*) and THY.AP5 (*MATα URA3*, *leu2*, *trp1*, *his3 loxP*::*ade2*) yeast strains were transformed with the pQdr2-Cub (Cub clones) and pNub-Erv14*^S134A^* or pNub-Erv14*^S134D^* (Nub clones) constructions, respectively. Cells were allowed to mate on YPD medium for 24 h at 30°C, and then, diploid cells were selected on SC medium. For testing interactions, serial dilutions of cells were applied on SC medium supplemented with 5 µM to 500 µM methionine, and cell growth was recorded for 3 days. The β-galactosidase activity of the lacZ (Fig. 3B), was revealed with 5-bromo-4-chloro-3-indolyl β-D galactoside (X-gal; Sigma-Aldrich, Carlsbad, CA, USA). Diploid cells were grown on SC medium supplemented with 20 mg/l adenine hemisulfate, 20 mg/l histidine-HCl and then covered with an X-gal–agarose layer (0.5% agarose, 0.5 M phosphate buffer, pH 7.0, 0.1% SDS and 0.6 mg/ml X-gal). Blue staining was developed for 1–24 h.

### Live-cell imaging

To analyze the subcellular localization of the GFP-tagged proteins, cells were grown in liquid YNB medium to mid-log phase. A portion (1 ml) of the cultures was harvested, centrifuged at 2795  ***g*** for 5 min using an GS15R table-top centrifuge (Eppendorf) and cell pellets resuspended in (25 µl) of YNB medium. 5 µl of resuspended cells were placed on a slide and were observed under an inverted multiphotonic confocal microscope (Olympus FV1000) equipped with a 60×1.3 NA oil immersion objective. GFP fluorescence was visualized by excitation with a multi-line Argon laser at 488 nm and spectral detector was set between 515/30 nm for emission. Images were processed with ImageJ (Schindelin et al., 2012).

### Preparation of semi-intact cells and membrane fractionation

To maintain selective pressure on the plasmids, yeast cells were grown overnight in selective medium YNB and diluted to an OD_600_ of 0.1 in 50 ml of YPD medium the following morning, and then grown to approximately an OD_600_ of 1.0 and harvested by centrifugation in an Sorvall RC 6 Plus centrifuge (1370 ***g***, 5 min, SS-34 rotor) at room temperature. The cell pellet was resuspended in buffer (100 mM Tris-HCl pH 9.4, 5 mM DTT), incubated for 10 min at room temperature and centrifuged as before. The cells were resuspended and converted into spheroplasts by digestion with (+)-lyticase buffer [20 mM Tris-HCl pH 7.5, 0.7 M sorbitol, 0.5% glucose, 2 mM DTT and 1 mg/ml lyticase (Sigma-Aldrich, Mexico; cat. 04963)]. The cells were incubated at room temperature until the OD_600_ of a 1:100 dilution in H_2_O was less than 80% of the initial value and then spheroplasted cells were diluted into ice-cold (−)-lyticase buffer (20 mM Tris-HCl pH 7.5, 0.7 M sorbitol, 0.5% glucose and 2 mM DTT) followed by centrifugation at 2795 ***g*** (Sorvall RC 6 Plus centrifuge with an SS-34 rotor) for 5 min at 4°C. Spheroplasts were resuspend in B88 buffer (20 mM HEPES pH 6.8, 250 mM sorbitol, 150 mM KOAc and 5 mM MgOAc), aliquoted, frozen in liquid nitrogen and stored at −80°C. Spheroplasts were used as a membrane source in the vesicle budding assay. Isolation of ER- and Golgi-enriched fractions followed a similar procedure. After converting into spheroplasts, pelleted cells were resuspended in 2.5 ml JR lysis buffer (20 mM HEPES pH 7.4, 0.1 M sorbitol, 50 mM KOAc, 2 mM EDTA, 1 mM DTT and 1 mM PMSF) and lysed with six strokes of a Dounce homogenizer and centrifuged in an SS-34 rotor at 447 ***g*** at 4°C for 5 min, to pellet nuclei and unbroken cells. The supernatant was recovered and centrifuged at 24,104 ***g*** for 10 min at 4°C in an Eppendorf 5417R centrifuge to generate the ER-enriched P13 pellet fraction. The supernatant was recovered and centrifuged at 100,000 ***g*** for 15 min at 4°C in an Optima TL micro-ultracentrifuge (TLA 100 rotor Beckman Coulter, Fullerton, CA) to obtain the Golgi-enriched p100 pellet fraction. The P13 and P100 pellets were resuspended in 100 µl JR lysis buffer and diluted 1:1 with 5× sample buffer (0.125 M Tris-HCl pH 6.8, 25% glycerol, 4% SDS and a trace of Bromophenol Blue), boiled, and separated on 12% SDS-PAGE gels. Immunoblotting was conducted with polyclonal antibodies against Yet3 (ER marker; 1:1000; Wilson and Barlowe, 2010) and Och1 (Golgi marker; 1:1000; Otte et al., 2001), that were used as fractionation controls; anti-GFP (Sigma-Aldrich, G1544, Lot 025M4754V) was used to monitor Erv14–GFP or Nha1–GFP.

### *In vitro* vesicle budding assay

The COPII vesicle budding assay was performed as previously described (Barlowe et al., 1994). Washed semi-intact cell (SICs) were obtained from cells expressing either HA-tagged wild type (Erv14) or the mutant versions (Erv14*^S134A^*) or (Erv14S*^134D^*) in BY4742*Δerv14* cells and incubated in the presence or absence of purified COPII proteins (Sar1, Sec23–Sec24 and Sec13–Sec31), GTP and an 10× ATP regeneration system, and incubated at 25°C for 30 min, for reconstitution of vesicle formation (Barlowe et al., 1994). A pool of 12.5 µl from reactions with and without COPII was mixed with 200 µl of B88 buffer, which represented 10% of the total reaction (T) that served as a loading control. SICs were pelleted at 24,104 ***g*** for 3 min at 4°C in an Eppendorf 5417R centrifuge. The supernatant containing vesicles was centrifuged at 100,000 ***g*** (Optima MAX-XP Benchtop Ultracentrifuge with a TLA100 rotor; Beckman Coulter, Fullerton, CA) to collect the membranes. Membrane pellets were resuspended in 25 µl of sample buffer 5×, and 8 or 10 µl aliquots were loaded on 14% polyacrylamide gels and immunoblotted (see below) for Sec61, Erv41, Coy1 or with anti-HA for Erv14–3HA (WT) or mutants Erv14*^S134D^*–3HA, Erv14*^S134A^*–3HA detection. To estimate packaging efficiency, the intensity of the bands was quantified using GeneTools image analysis software (Syngene), where (T) represents 10% of the reaction and was used to compare that from the vesicles obtained from the different Erv14 isoforms.

### Erv14 phosphoproteomic analysis by tandem mass spectrometry

For sample preparation, three independent microsomes replicates (10 µg per replicate) were run on an SDS-PAGE. Protein spots from the gel that corresponded to the bands recognized by the HA antibody were sent to the Proteomics Facility at the Institut de Recherches Cliniques de Montréal, Canada. The samples were analyzed using Mascot software (Matrix Science, London, UK). Mascot was set up to search the Uniprot_S_cerevisiae_txid_4932 database. Scaffold (version Scaffold_4.2.1, Proteome Software Inc., Portland, OR) was used to validate LC-MS/MS-based peptide and protein identifications. Scaffold parameters were set to a minimum of two peptides per protein and accepted if they could be stablished with minimum probabilities of 99% at the protein level by the Peptide Prophet algorithm.

### SDS-PAGE and protein immunoblotting

Protein for Western blot analysis was precipitated by dilution of the samples 50-fold in 1:1 (v/v) ethanol:acetone and incubated overnight at −30°C as described (Parry et al., 1989). Samples were then centrifuged at 13,000 ***g*** for 20 min at 4°C using an F2402 rotor in a GS15R table-top centrifuge (Eppendorf). Pellets were air dried, resuspended with 4× SDS-PAGE sample buffer (125 mM Tris-HCl pH 6.8, 25% glycerol, 4% SDS, 5% β-mercaptoethanol and Bromophenol Blue), and heated at 95°C for 5 min before loading onto 12% (w/v) linear acrylamide mini-gels. For vesicle budding assay samples, pellets were resuspended in sample buffer 4×, boiled at 95°C for 5 min, and resolved onto 14% (w/v) SDS-PAGE gels. After protein separation, gels were either fixed and stained with Coomassie Brilliant Blue R 250 or electrophoretically transferred onto nitrocellulose membranes (Millipore) by standard methods. Following transfer, proteins were stained with Ponceau S protein stain (0.1% w/v in 1% v/v acetic acid for 30 s) to check for equal loading/transfer of proteins. Membranes were then blocked with TBS (100 mM Tris-HCl pH 7.5, 150 mM NaCl) containing 0.02% (w/v) Na-azide and 5% (w/v) fat-free milk powder (Nestle, Mexico) for 2 h at room temperature. Blocked membranes were incubated overnight with the appropriate primary antibodies, anti-HA (Sigma-Aldrich, H6908, lot # 098M4812V), anti-phospho-serine (Sigma-Aldrich, P3430, lot # 114M470V), anti-GFP (Sigma-Aldrich, G1544, lot # 025M4754V), anti-Erv41 and anti-Och1 (both described in Otte et al., 2001) or anti-Sec61 (Stirling et al., 1992). Primary antibodies were used at 1:1000 dilutions except for anti-HA, which was used at 1:6000 dilutions. For secondary antibodies, horseradish peroxidase-linked anti-rabbit-IgG and anti-mouse-IgG antibodies (GE Healthcare) were used at 1:10,000 dilutions. Blots were developed with two different reagents, chemiluminescent Luminata™ Crescendo procedure (Millipore) or SuperSignal West Pico Chemiluminescent substrate (Thermo Fisher Scientific, Waltham, MA, USA). Images were captured using either Gel DOC™ XR+ SYSTEM (BIORAD) or G:Box Chemi XR5 (Syngene). Full images of blots shown in this paper are available as Fig. S9.

### Analysis of phosphoproteins by Zn(II)-Phos-Tag SDS-PAGE

Phos-Tag SDS-PAGE protein separation was performed according to manufacturer's specifications (Wako Pure Chemical Industries). The Phos-tag gel was prepared with 10% acrylamide and 50 µM Phos-tag as described below. For the resolving gel, 2.67 ml of solution [30% acrylamide-bis, 2.5 ml of 1.5 M Tris-HCl pH 8.8, 100 µl of 5 mM Phos-tag (Wako Pure Chemical Industries), 100 µl of 10 mM ZnCl_2_, 100 µl of 10% SDS, 10 µl TEMED, 25 µl of 10% APS and 4.5 ml of distilled water] was used. The stacking gel was prepared with 600 µl of 30% acrylamide-bis-acrylamide (Sigma-Aldrich, Carlsbad, CA, USA), 1 ml of 0.5 M Tris-HCl pH 6.8, 40 µl of 10% SDS, 4 µl TEMED, 20 µl of 10% ammonium persulfate and 2.34 ml of distilled water. The gel was run at 30 mA for 6 h at 4°C. After electrophoresis, the resolving gel was washed three times in 100 ml of transfer buffer (192 mM glycine, 25 mM Tris base pH 8.0, and 10% methanol containing 10 mM EDTA) and then washed once for 20 min in transfer buffer without EDTA. Protein was blotted onto nitrocellulose membrane at 30 V (at constant voltage) for 8 h at 4°C. Membranes were blocked using 5% nonfat milk and probed with 1:10,000 dilution of anti-HA (Sigma-Aldrich, H6908, lot # 098M4812V).

### Electron microscopy

For transmission electron microscopy, samples were prepared as described previously (Wright, 2000). Yeast cells were grown in YNB selective medium at mid-log phase (OD_600_ 0.8). Cells were centrifuged at 1500 ***g*** for 5 min in an F2402 rotor in a GS15R table-top centrifuge (Beckman) and resuspended in water to wash the cells. The pellet was fixed in ice-cold fixing cacodylate buffer (0.1 M sodium cacodylate, pH 6.8, 1 mM MgCl_2_, 1 mM CaCl_2_ and 2% glutaraldehyde) for 2 h at 4°C and centrifuged as above. Cells were resuspended in cacodylate buffer and incubated overnight at 4°C. Cells were pelleted as above and washed three times in cacodylate buffer; then, cells were resuspended in 2% KMnO_4_ for 1 h at room temperature. After secondary fixing, cells were resuspended in 1 ml of freshly prepared 2% uranyl acetate for 1 h at room temperature and subsequently washed in water. Dehydration of cells was followed by clearing with propylene oxide and infiltration with a combination of 100% propylene oxide and 100% Spurr low-viscosity resin (TED PELLA.INC, USA). Finally, samples were embedded in 100% resin and kept in a vacuum oven at 45°C for 12 h, then shifted to 68°C for 3 days. For TEM examination, thin sections of grey-silver color interference (60–70 nm) were cut and mounted onto 300-mesh copper grids. The mounted sections were stained with alkaline lead citrate, washed gently with distilled water and allowed to dry for 1 h. Dried sections were observed using a CARL ZEISS Libra 120 (Carl Zeiss AG, Oberkochen, Germany) transmission electron microscope, at a voltage of 80 kV, coupled to a Gatan Ultrascan 1000 CCD (2000×2000 pixels) (MultiScan camera model 794 gatan, Pleasanton, California, USA) to record the images.

## Supplementary Material

10.1242/joces.260527_sup1 Supplementary information
