# Supplementary material for: The C-terminus of the cargo receptor Erv14 affects COPII vesicle formation and cargo delivery
Source: J Cell Sci. 2023 Feb 6;136(3):jcs260527. doi: 10.1242/jcs.260527 (PMC10022740; doi:10.1242/jcs.260527)
Supplement: Supplementary information [file joces-136-260527-s1.pdf]

A

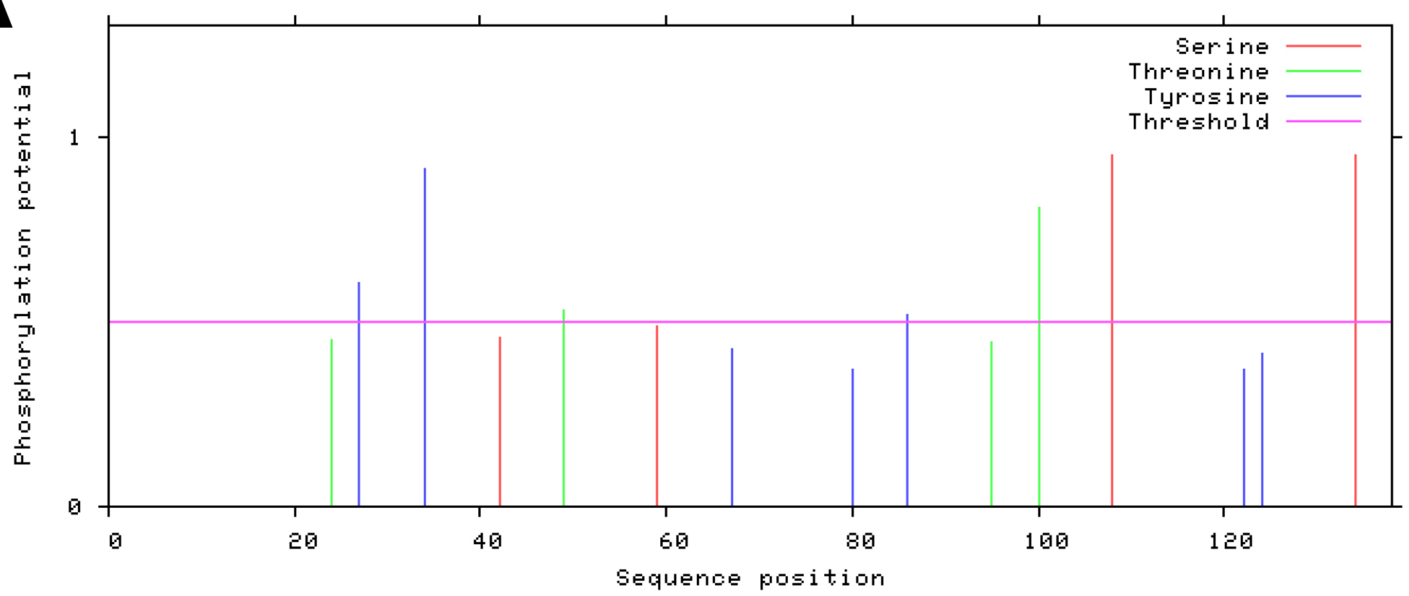

B

#Aminoacid	Context	Score
108S	HKRESFLKL	0.949
134S	LIAESGDDF	0.948

Fig. S1. Search of putative phosphorylation sites using Netphos 3.1.
A). The plot shows predicted phosphorylation sites in the Erv14 sequence. **B).** The two possible phosphorylatable Serine residues and their sequence consensus.

Journal of Cell Science • Supplementary information

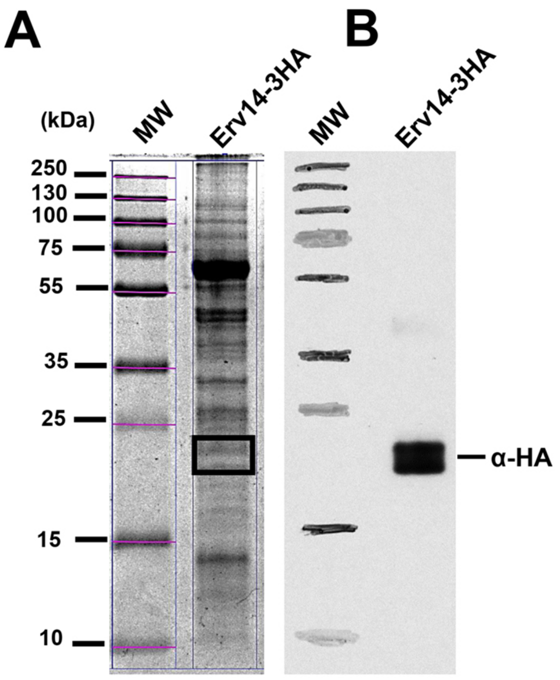

C

Sample	Protein Name	Protein MW (kDa)	Unique Peptide Count	Unique Spectra Count	Total Ion Spectra
1	Erv14	16	2	3	9
2	Erv14	16	2	2	6
3	Erv14	16	2	2	6

D

Calc. Mass (Da)	Start Seq.	End Seq.	Sequence	Ion Score
1822.00	85	99	(K)IYNKVQLLDATEIFR(T)	30.7
1303.71	89	99	(K)VQLLDATEIFR(T)	31.6

Fig. S2. Search for phosphorylation sites by LC-MS/MS. A) SDS-PAGE gel showing the selected area (square). **B)** Western blot developed with (α-HA) antibody. **C)** Table showing the number of peptides identified and the number of total spectra obtained by LC-MS/MS. **D)** Properties of the identified peptides.

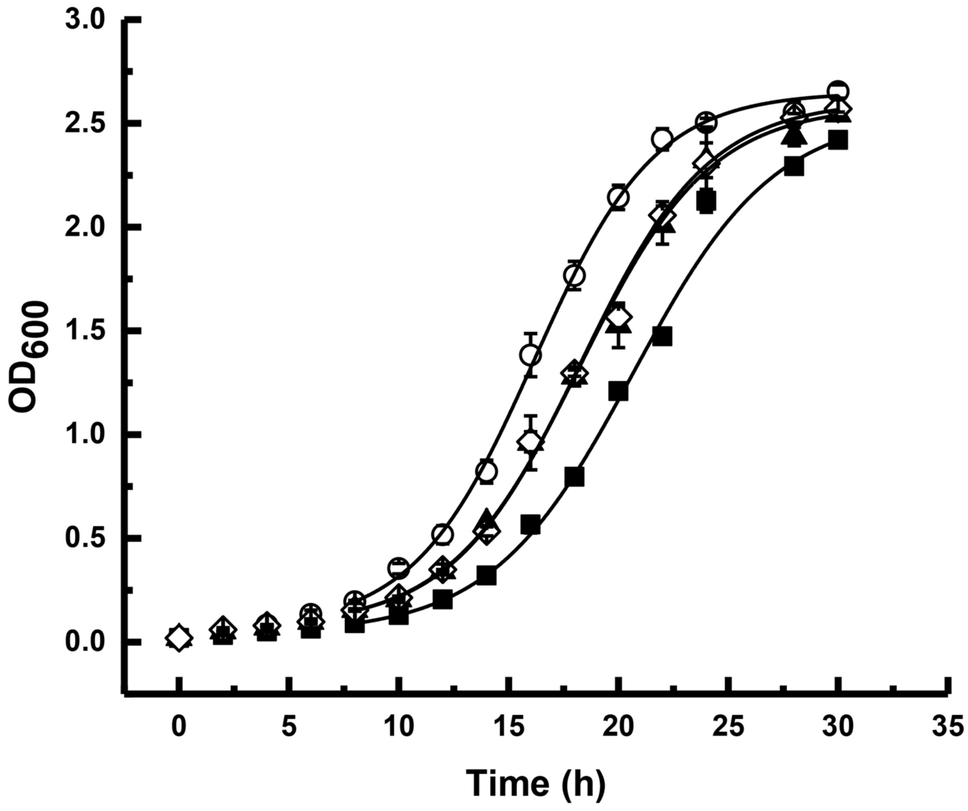

Fig. S3. Cell growth curve from BY4741*erv14Δ* strain expressing wild type Erv14 and mutants. (○) Wild type Erv14, (▲) Dephosphorylated form Erv14^{S134A}, (◊) Phosphomimetic form Erv14^{S134D}, (■) Empty vector pDR-F1.

Journal of Cell Science • Supplementary information

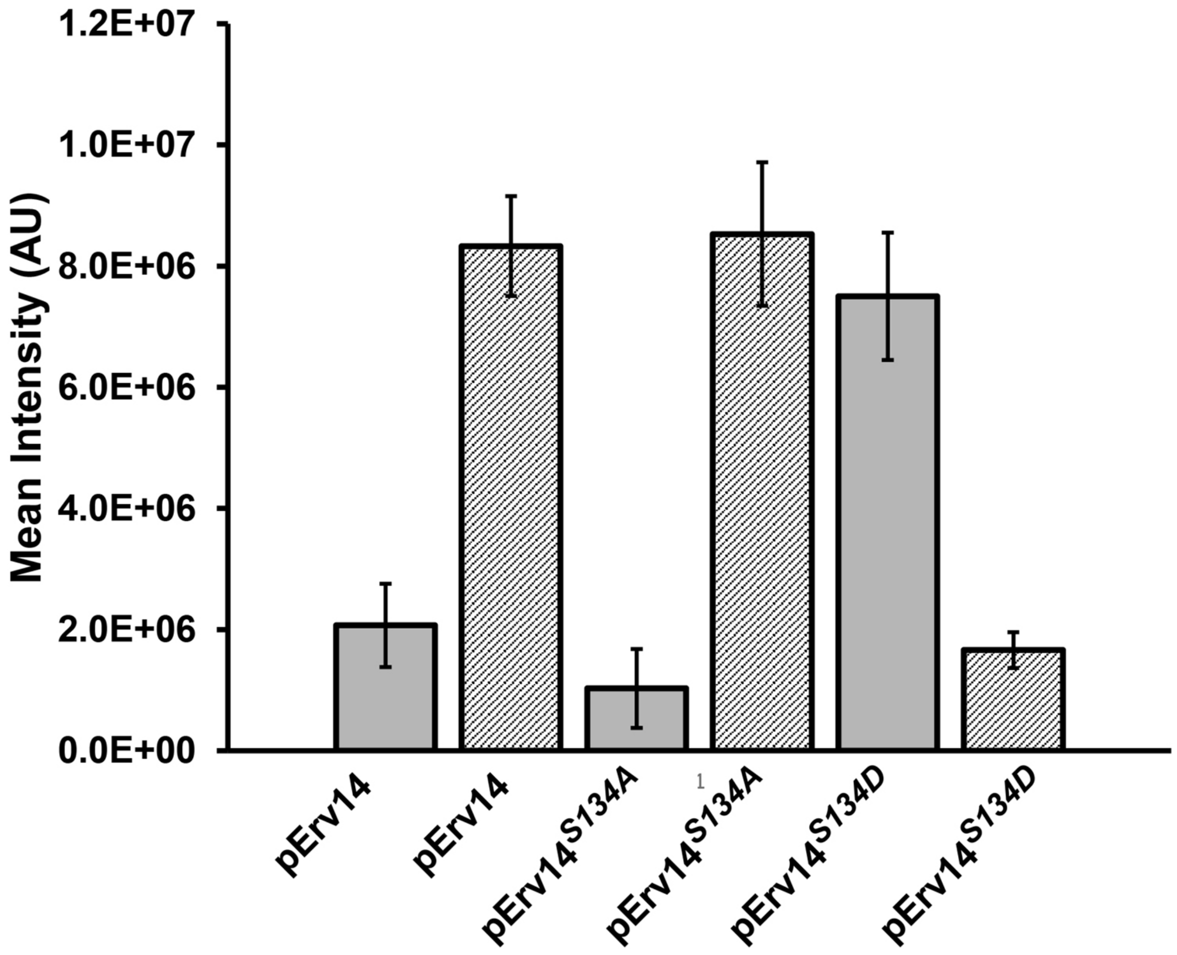

Fig. S4. Quantification of band intensities from Figure 2B. Intensities from the bands corresponding to the recognition of Nha1-GFP in the ER (grey bars) or PM/GA (hatched bars) by the anti-GFP antibody from yeast cells co-transformed with the *ERV14* or the corresponding mutant. Data are the mean ± SD from three different preparations.

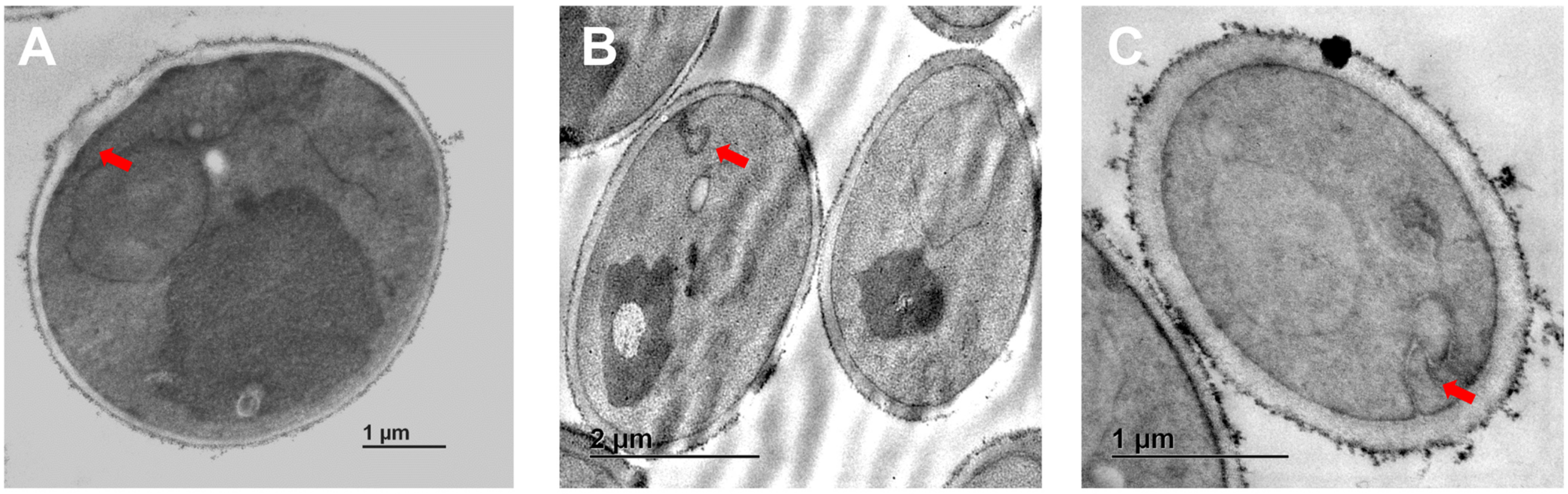

D

ER deformation	pERV14	pERV14^{S134A}	pERV14^{S134D}	*erv14Δ*
Absence	17	6	8	14
Presence	1	16	21	3
Total	18	22	29	17
t-test *p*-values		0.000447238	0.001756611	0.37390097

Fig. S5. Mutations at Ser-134 modifies ER structure. TEM micrographs from BY4741erv14Δ cells expressing Erv14 or the mutants Erv14S134A, or Erv14S134D. **A)** In Erv14p expressing cells the cortical ER was observed as a continuous thread closely attached to the PM. In cells expressing the mutants Erv14S134A (red arrow) **(B)** or Erv14^{S134D} **(C)**, cortical ER showed deformation in omega like structures. **(D)** Table showing the number of cell with omega-like or without ER modification. The t-test Student analysis was used to compare each mutant to wild type with p values: ns, p>0.05, significant p<0.05, highly significant p<0.001.

Journal of Cell Science • Supplementary information

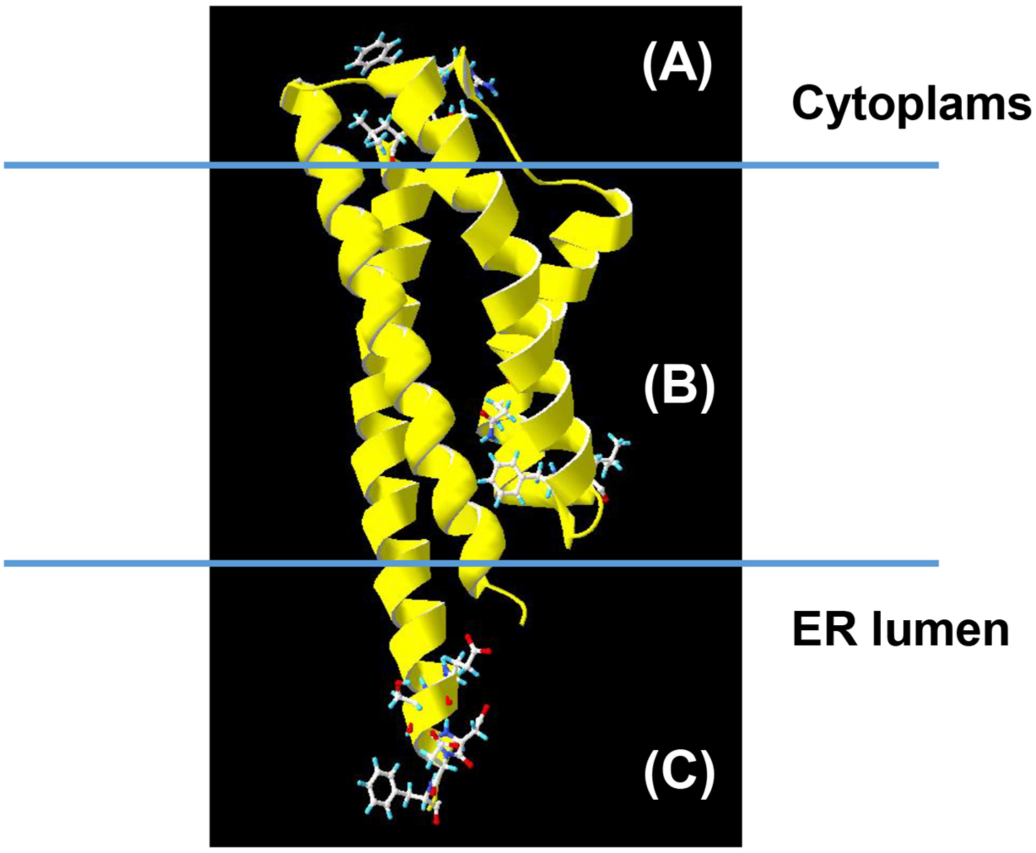

Fig. S6. Erv14/cornichon molecular model obtained with the Alphafold2 algorithm. Erv14 was predicted to possess four membrane spanning helices with the C and N termini facing the ER lumen. **A**) The COPII-binding site (IFRTL); **B**) Cargo-binding site; **C**) Possible phosphorylation domain. The position of the membrane limits is approximate

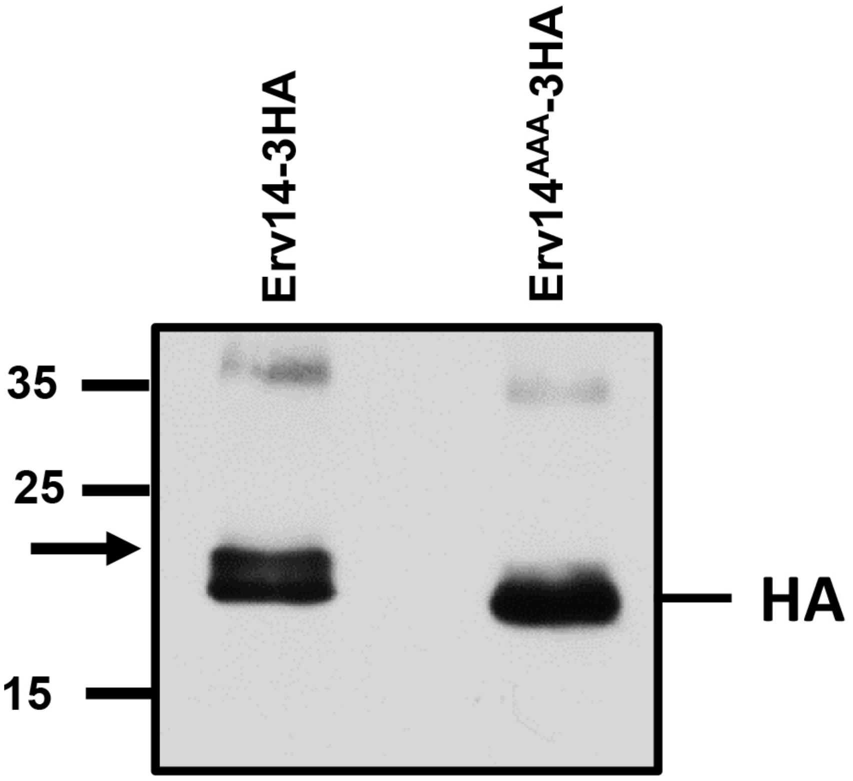

Fig. S7. Mutations on the phosphorylation domain prevent the modification of Erv14. Blot from the P13 fraction showed an additional band in the WT protein (Erv14-3HA; arrow) that disappeared by elimination of the acidic amino acids required for the establishment of the phosphorylation domain (Erv14^{AAA}-3HA).

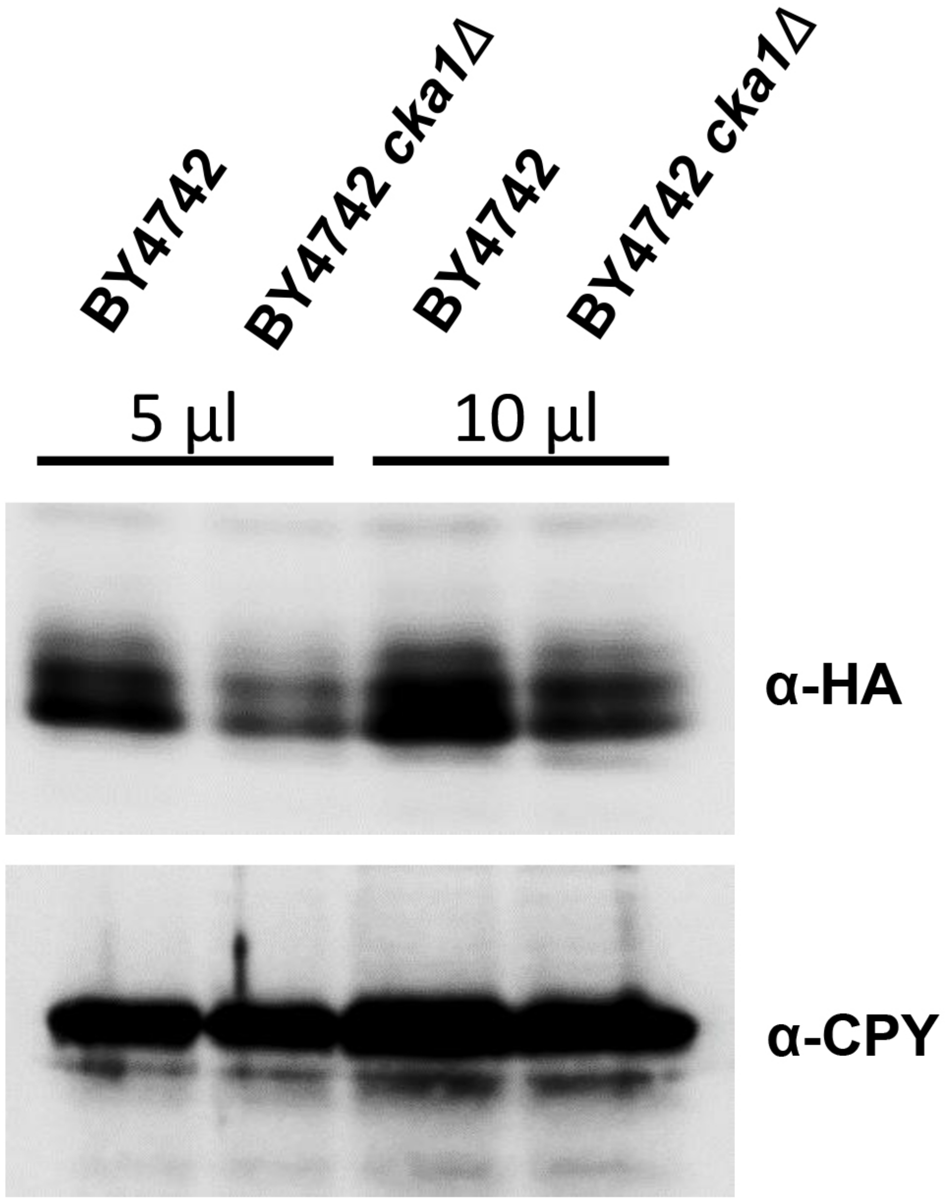

Fig. S8. Analysis of Erv14 isoforms on the *ck1Δ* mutant. Western blot demonstrating the abundance of Erv14-3HA in the microsomal fraction from BY4742 or BY4742*cka1Δ* cells. Observe the lower abundance of Erv14 when expressed in BY4742*cka1Δ*. CPY was used as a loading control.

Journal of Cell Science • Supplementary information

Fig. S9. Blot and data transparency

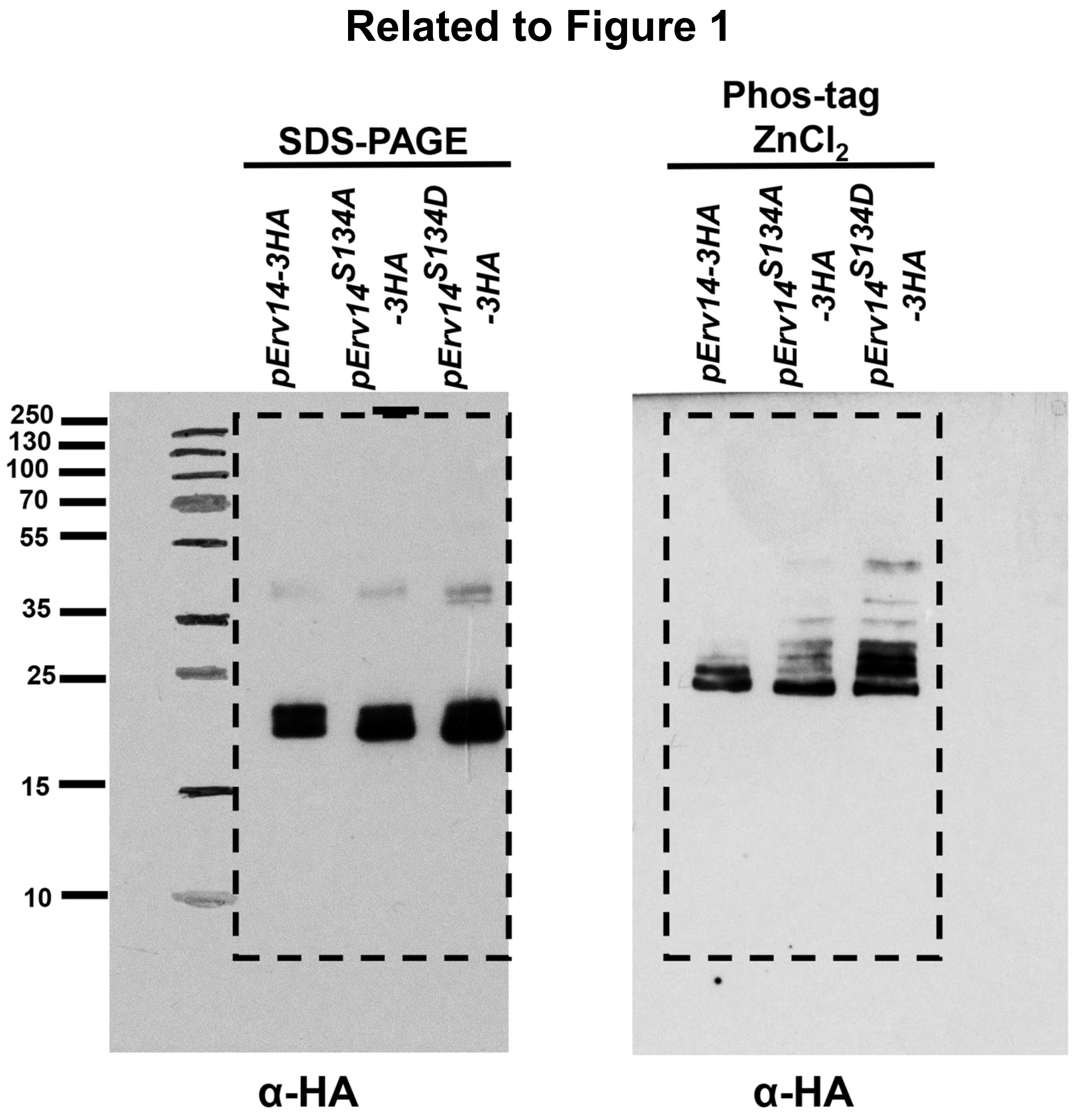

Blot Transparency: Blots from HA-tagged Erv14, Erv14^{S134A} or Erv14^{S134D} expressing cells were separated in SDS-PAGE (left) or Zn(II)-Phos-tag (right) gels. Anti-HA antibody (19 kDa) was used to developed. Dashed inset indicated the cut region used in Figure 7

Journal of Cell Science • Supplementary information

Related to Figure 2

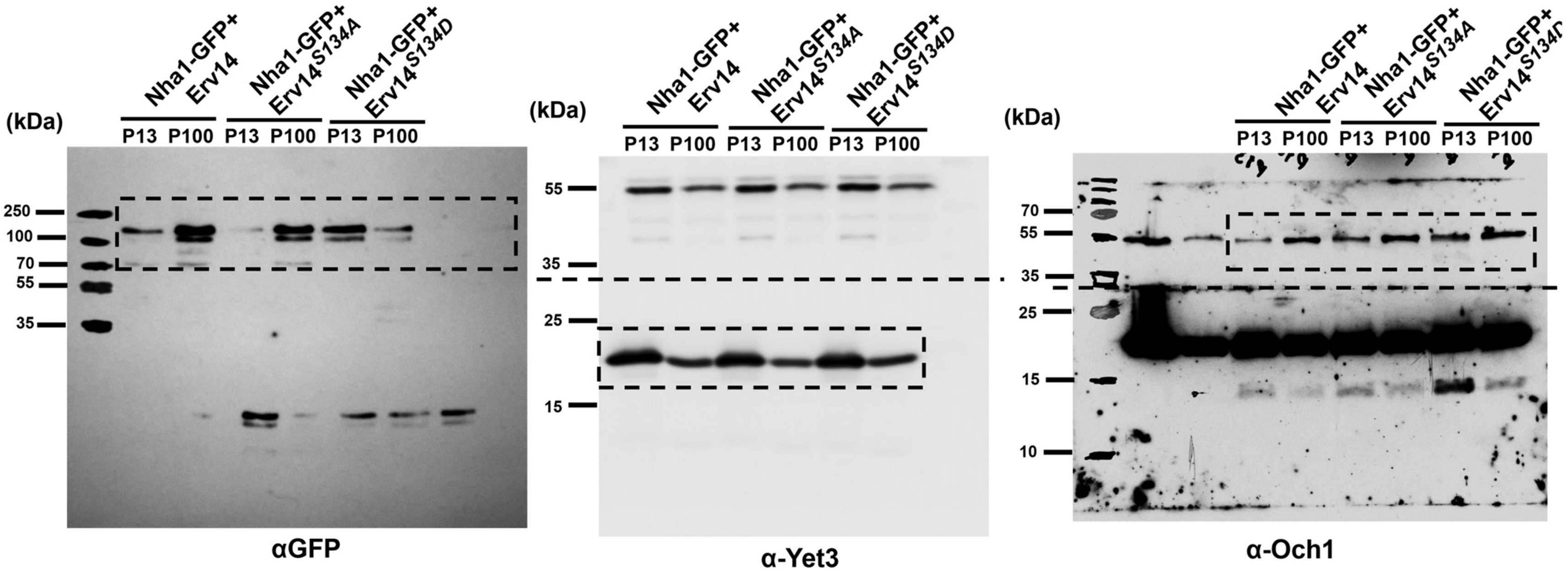

Blot Transparency: Blots from plasma membrane localization of the Nha1 exchanger in BYT45*erv14Δ* cells transformed with Wild type pERV14 and point mutations. Immunoblotting was conducted with Anti-GFP to monitor Nha-GFP (130 kDa). Yet3 (P13) and Och1 (P100) were used as fractionation controls. Dashed line and inset indicated cut regions shown in Figure 1.

Journal of Cell Science • Supplementary information

Data Transparency

Related to Figure 4

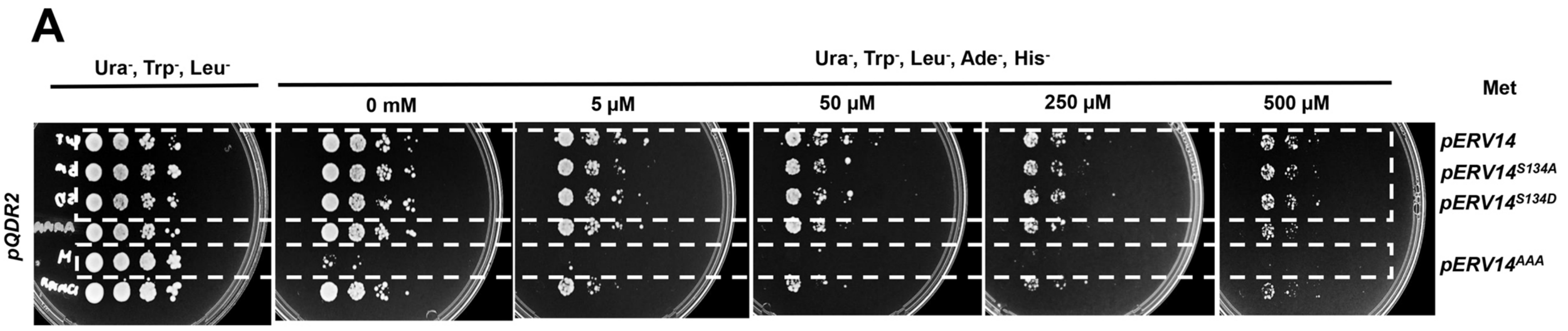

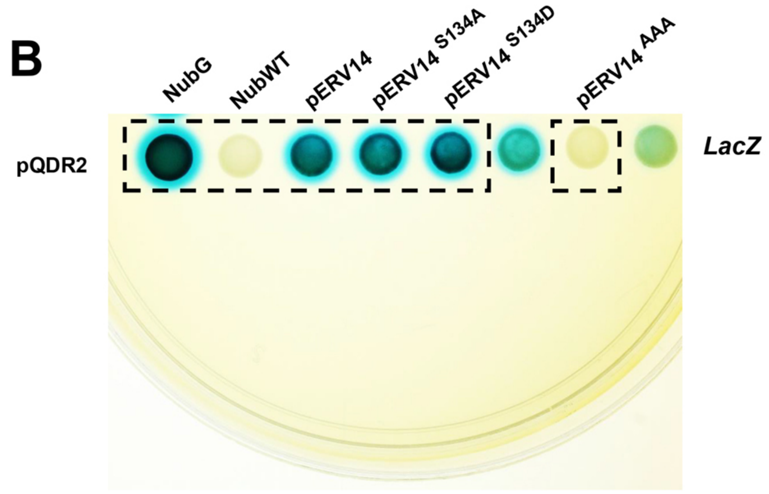

Original images from the mbSUS (A) and LacZ (B) assys. Broken lines enclose the interactions between QDR2 and the Erv14 mutants indicated.

Journal of Cell Science • Supplementary information

Related to Figure 5

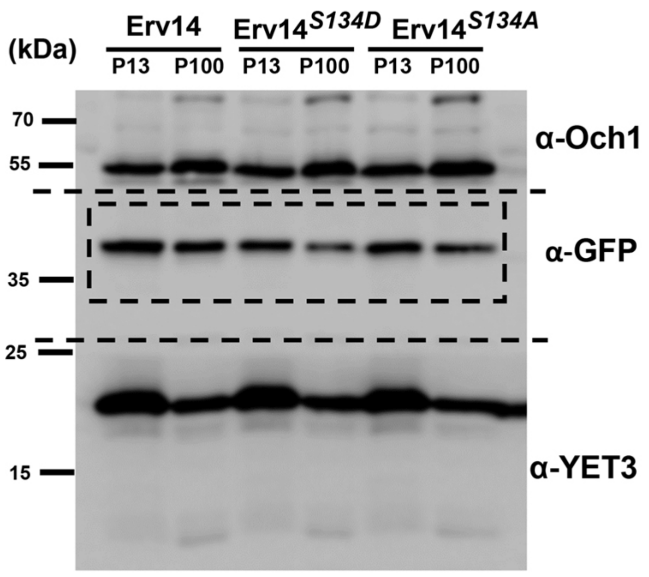

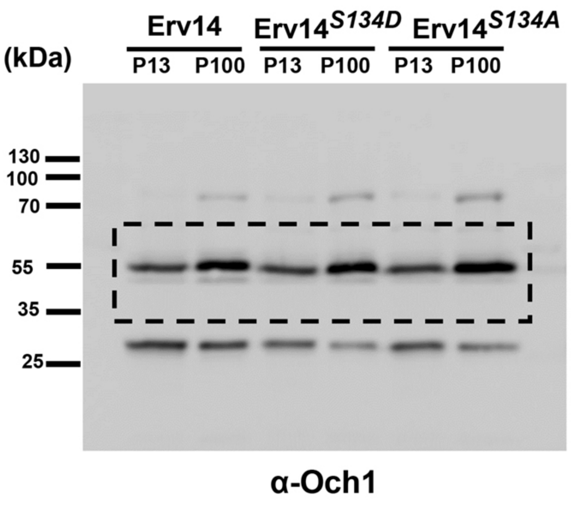

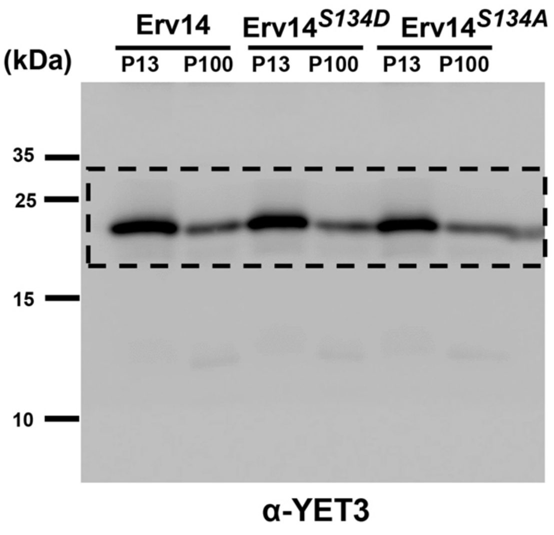

Journal of Cell Science • Supplementary information

Table S1. List of strains used in this study

Strain	Genotype	Source
BY4741Δerv14	MATa his3Δ1 leu2Δ0 met15Δ0 ura3Δ0 erv14Δ::KanMx	Our Lab
BY4741Δerv14Δpdr12	MATa his3Δ1 leu2Δ0 met15Δ0 ura3Δ0 erv14Δ::loxP pdr12Δ::KanMx	Our Lab
BY4741Δerv14Δqdr2	MATa his3Δ1 leu2Δ0 met15Δ0 ura3Δ0 erv14Δ::loxP qdr2Δ::KanMx	Our Lab
BYT45erv14Δ	MATa his3Δleu2Δmet15Δura3Δ; nha1Δ::loxP ena1-5Δ::loxP erv14Δ::KanMx	Our Lab
BY4742Δerv14	MATα his3Δ1 leu2Δ0 lys2Δ0 ura3Δ0 erv14Δ::KanMx	Barlowe Lab
CBY617 (FY834)	MATα his3Δ200 ura3-52 lys2Δ202 trp1Δ63 erv14::HIS3 sec23-1	Barlowe Lab
CBY663 (FY834)	MATα his3Δ200 ura3-52 leu2Δ1 lys2Δ202 erv14::HIS3 sec16-2	Barlowe Lab
CBY603 (FY834)	MATα his3Δ200 ura3-52 leu2Δ1 lys2Δ202 trp1Δ63 erv14::HIS3 sec13-1	Barlowe Lab
THY.AP4	MATa ura3 leu2 lexA::lacZ::trp1 lexA::HIS3 lexA::ADE2	Frommer Lab
THY.AP5	MATα URA3 leu2 trp1 his3 loxP::ade2	Frommer Lab
BW31a	*MATa leu2-3/122 ura3-1 trp1-1 his3-11/15 ade2-1 can1-100 GAL SUC2 mal10 ena1-4Δ::HIS3 nha1::LEU2*	Our Lab

Journal of Cell Science • Supplementary information

Table S2. Primers used for gene cloning into the Gateway vectors and pGRU1

	5’	3´
**Gene**	**Forward**	**Reverse**
**ERV14^{S134A}-pDONOR221**	GTACAAAAAAGCAGGCTTCATGGGTGCTTGGTTATTTATC	GTACAAGAAAGCTGGGTCGAAGTCATCACCAGCTTCAGCAATC
**ERV14^{S134D}-pDONOR207**	GTACAAAAAAGCAGGCTTCATGGGTGCTTGGTTATTTATC	GTACAAGAAAGCTGGGTCGAAGTCATCACCATCTTCAGCAATC
**ERV14^{S134A}-pGRU1**	GTACATTATAAAAAAAAATCCTGAACTTAGCTAGATATTATGGGTGCTTGGTTATTTATCC	TAAAGCTCCGGAGCTTGCATGCCTGCAGGTCGACTCTGAAGTCATCACCAGCTTCAGCAATC
**ERV14^{S134D}-pGRU1**	GTACATTATAAAAAAAAATCCTGAACTTAGCTAGATATTATGGGTGCTTGGTTATTTATCC	TAAAGCTCCGGAGCTTGCATGCCTGCAGGTCGACTCTGAAGTCATCACCATCTTCAGCAATC

Journal of Cell Science • Supplementary information
