# Supplementary material for: The C-terminus of the cargo receptor Erv14 affects COPII vesicle formation and cargo delivery
Source: J Cell Sci. 2023 Feb 6;136(3):jcs260527. doi: 10.1242/jcs.260527 (PMC10022740; doi:10.1242/jcs.260527)
Supplement: Supplementary file 1 [file joces-136-260527_review_history.pdf]

Journal of Cell Science

The C-terminus of the cargo receptor Erv14p affects COPII vesicle formation and cargo delivery

Daniel Gomez-Lagunas, Carolina Yañez-Dominguez, Guadalupe Zavala-Padilla, Charles K Barlowe and Omar Pantoja

DOI: 10.1242/jcs.260527

Editor: Jennifer Lippincott-Schwartz

Review timeline

Original submission:	16 August 2022
Editorial decision:	7 November 2022
First revision received:	23 November 2022
Accepted:	22 December 2022

Original submission

First decision letter

MS ID#: JOCES/2022/260527

MS TITLE: The C-terminus of the cargo receptor Erv14p affects COPII vesicle formation and cargo delivery

AUTHORS: Daniel Gomez-Lagunas, Carolina Yanez-Dominguez, Guadalupe Zavala-Padilla, Charles K Barlowe, and Omar Pantoja
ARTICLE TYPE: Research Article

We have now reached a decision on the above manuscript.

To see the reviewers' reports and a copy of this decision letter, please go to: https://submit-jcs.biologists.org and click on the 'Manuscripts with Decisions' queue in the Author Area. (Corresponding author only has access to reviews.)

As you will see, the reviewers raise a number of substantial criticisms that prevent me from accepting the paper at this stage. They suggest, however, that a revised version might prove acceptable, if you can address their concerns. If you think that you can deal satisfactorily with the criticisms on revision, I would be pleased to see a revised manuscript. We would then return it to the reviewers.

Please ensure that you clearly highlight all changes made in the revised manuscript. Please avoid using 'Tracked changes' in Word files as these are lost in PDF conversion.

I should be grateful if you would also provide a point-by-point response detailing how you have dealt with the points raised by the reviewers in the 'Response to Reviewers' box. Please attend to all of the reviewers' comments. If you do not agree with any of their criticisms or suggestions please explain clearly why this is so.

Reviewer 1

Advance summary and potential significance to field

© 2023. Published by The Company of Biologists under the terms of the Creative Commons Attribution License (https://creativecommons.org/licenses/by/4.0/).

This manuscript from the Pantoja lab identifies a putative phosphorylation site on the conserved ER export receptor, Erv14, that seems to influence not only Erv14 traffic, but also more broadly impacts ER export more globally with follow-on effects on ER morphology, cell growth etc. Generally, the data are solid and use appropriate experimental evidence to support the model that Erv14 capture into COPII vesicles is impacted rather than client engagement. That said, there are some flaws in some of the interpretations.

Comments for the author

• The authors should be more up-front about the location of the putative phosphorylation site on the lumenal side of the membrane. To my knowledge there is not a lumenal kinase that is known to participate in ER-Golgi traffic. Some speculation about the identity of such a yeast enzyme seems warranted. Alternatively, the possibility that the mutations might indirectly impact phosphorylation or structure of cytoplasmic domains should be entertained.

• For Figure 3, it seems appropriate to include a positive control for the split-ubiquitin assay where interaction with the cargo is in fact disrupted. The previously reported Erv14-AAA mutant on the same plates would reassure that the selection is working as expected.

• Without wanting to re-write the author's manuscript, we were left thinking that Figure 6 might be more appropriate as Figure 1, since the mass spectrometry didn't confirm the PTM of S134, the evidence in Figure 6 that support that mutations impact phosphorylation state of the protein to motivate the study makes a bit more sense.

Reviewer 2

Advance summary and potential significance to field

In the work, the authors attempted to address how post-translational modification specifically phosphorylation, of Erv14 cargo receptor affects secretory pathways. They've applied a battery of assays to demonstrate that the synthetic, phospho-mimetic Erv14-S134D abolished cargo trafficking, Erv14 localization, and COPII vesicle formation. All the experiments were conducted with controls and the results were interpreted properly. All the data indicated that mutations in the C' of Erv14 affected secretory pathway. However, the main issue of this work is its physiological relevance there is no evidence of Erv14 phosphorylation at S134 in vivo. This significantly diminishes the claims of this work. In my opinion, filing this gap is required for publication in JCS.

Comments for the author

The major issues must be addressed for publication:

1. Physiological relevance – if Erv14 can be phosphorylated inside cells? As the authors stated that phospho-mimetic Erv14 can affect the secretory pathway, there is no definite evidence indicating that Erv14 can be phosphorylated in vivo. The phos-tag SDS-PAGE assays suggested possible phosphorylation in Erv14. The authors could take the advantage of this assay to further characterize/confirm Erv14 phosphorylation. Conditions such as activation or knockdown of CK2 could be applied as controls. Definite experimental evidence of Erv14 phosphorylation must be shown.

2. Phos-tag assay suggested potential phosphorylation sites in Erv14-S134A/D mutants (slower migrating bands) because S134 site was mutated and could not be phosphorylated. The authors at least need to address this in the text. A related issue is the contradictory data from mass-spec analysis, as no modifications were detected. These inconsistences must be resolved as they are key to the main claim of this work.

3. The results of synthetic mutation experiments are confusing (Figure 6C). In Sec23-1 erv14Δ strain, reconstitution of Erv14 rescued growth defects. However, opposite results, i.e. further

© 2023. Published by The Company of Biologists under the terms of the Creative Commons Attribution License (https://creativecommons.org/licenses/by/4.0/).

inhibition of growth, were shown in Sec16-2 erv14∆ and Sec13-1 erv14∆ strains. As Sec23, Sec13, and Sec16 primarily involve in the same pathway, this inconsistence needs to be reconciled.

4. Data of ER structural changes caused by Erv14 mutations are weak (figure 5). Only one low-mag EM image was shown in each condition. High-mag images and quantification of these changes are required to support the conclusion. In addition, it would be nice to see the secretory compartments in these conditions using EM, which will be synergistic to vesicle formation results (Figure 6A).

First revision

Author response to reviewers' comments

Dear Dr. Lippincott-Schwartz,

We acknowledge the comments made by the Reviewers that will help to improve our manuscript. Here, we give an answer to different points raised by each Reviewer highlighting those to Reviewer 1 in yellow and for Reviewer 2, in red text.

Reviewer 1 Comments for the Author...

• The authors should be more up-front about the location of the putative phosphorylation site on the lumenal side of the membrane. To my knowledge there is not a lumenal kinase that is known to participate in ER-Golgi traffic. Some speculation about the identity of such a yeast enzyme seems warranted. Alternatively, the possibility that the mutations might indirectly impact phosphorylation or structure of cytoplasmic domains should be entertained.

In our manuscript we clearly describe the localization of the C-terminus, that includes Ser134, as located in the lumen of the ER. Additionally, we obtained the molecular structure with the AlphaFold algorithm that clearly shows that Erv14 possesses four transmembrane domains with both, N- and C termini, facing the ER lumen. Moreover, and in agreement with the Reviewer's comment on the lack of any evidence on the presence of ER located kinases, we propose that phosphorylation of Ser134 occurs at the Golgi lumen, where several kinases have been identified, among them, family with sequence similarity 20C (Fam20C), and Hrr25 that seems to phosphorylate the COPII subunits Sec23/Sec24. Additionally, and again in agreement with the Reviewer on possible structural modifications caused by the phosphorylation of Erv14, in the Discussion we stated that "It is possible that by simulating phosphorylation of S134, a conformational change in Erv14 is induced, precluding its correct interaction with the Sec23/Sec24 complex, and therefore, the formation of COPII vesicles." Based on our results, we are now adding Fig. 8 to propose a pathway to highlight the importance of the C-terminus of Erv14 and the need for the phosphorylation of S134 to occur at the Golgi apparatus, activity that would give directionality to the COPII-mediated transport.

• For Figure 3, it seems appropriate to include a positive control for the split- ubiquitin assay where interaction with the cargo is in fact disrupted. The previously reported Erv14-AAA mutant on the same plates would reassure that the selection is working as expected.

Following the Reviewer's comment, we have modified old Figure 3, now Figure 4 to show that the interaction of Erv14 and the cargo protein Qdr2 is disrupted by elimination of the acidic amino acids at the C-terminus of Erv14. Results from both, cell growth and LacZ activity, from the different protein-protein interactions agree, corroborating that changes in the phosphorylation state of Erv14 does not seem to be important for the interaction with the cargo protein. We have added a brief description to this figure in the Results and Discussion sections. We would like to make clear that both Figures are the same. In the original Fig. 3, we removed parts that are not related to this work and the results from the Erv14-^{AAA} mutant. In new Fig. 4, we have done the same, but had to cut and paste

© 2023. Published by The Company of Biologists under the terms of the Creative Commons Attribution License (https://creativecommons.org/licenses/by/4.0/).

the results from the Erv14-^{AAA} mutant, as in the original figure these results were two rows below. Now we are including within the Blot Transparency supplemental information the original figures for both, the mbSUS and the LacZ assays.

• Without wanting to re-write the author's manuscript, we were left thinking that Figure 6 might be more appropriate as Figure 1, since the mass spectrometry didn't confirm the PTM of S134, the evidence in Figure 6 that support that mutations impact phosphorylation state of the protein to motivate the study makes a bit more sense.

Following the Reviewers suggestion, we have moved Figure 7, "Erv14 is phosphorylated at the carboxyl terminus" as Fig. 1 to give initial evidence that this protein modification does occur in Erv14.

Reviewer 2 Comments for the Author...

The major issues must be addressed for publication:
1. Physiological relevance – if Erv14 can be phosphorylated inside cells? As the authors stated that phospho-mimetic Erv14 can affect the secretory pathway, there is no definite evidence indicating that Erv14 can be phosphorylated in vivo. The Phos-tag SDS-PAGE assays suggested possible phosphorylation in Erv14. The authors could take the advantage of this assay to further characterize/confirm Erv14 phosphorylation. Conditions such as activation or knockdown of CK2 could be applied as controls. Definite experimental evidence of Erv14 phosphorylation must be shown.

In disagreement with the Reviewer, in new Fig. 1 (old Fig. 7) we show in the Phos-tag gel, the presence of an additional band in the WT protein, that upon mutation to an unphosphorylated isoform (S134A) it disappeared, confirming that the upper band observed together with the WT protein very likely corresponds to a modified isoform of Erv14. Confirmation of this result is the presence of a similar band in the phosphomimetic isoform (S134D). Additionally, we are now including Fig. S7, where absence of the upper band was observed in a different context, namely, by removal of the acidic amino acids that are required to establish the phosphorylation domain, without changing S134. These two results complement our interpretation that S134 is indeed phosphorylated. As indicated by the Reviewer, we also investigated the possibility that mutation of CK2 could modify the phosphorylation status of Erv14, however, and as we showed in Fig. S8, mutation of CKA1, the catalytic subunit of CK2, significantly reduced the abundance of Erv14, indicating a possible pleiotropic effect, as CK2 is known to regulate an important number of proteins. These results prevented us from obtaining direct evidence on the possible phosphorylation of Erv14 by CK2.

2. Phos-tag assay suggested potential phosphorylation sites in Erv14- S134A/D mutants (slower migrating bands) because S134 site was mutated and could not be phosphorylated. The authors at least need to address this in the text. A related issue is the contradictory data from mass-spec analysis, as no modifications were detected. These inconsistences must be resolved as they are key to the main claim of this work.

In the manuscript we stated "The detection of additional bands with the S134D mutant may be due to a strong interaction between the carboxylate group from aspartate, and the Zn^{+2}-Phos-tag complex" addressing the concern from the Reviewer. Although Erv14S134D can't be phosphorylated, it possesses the carboxylate group that by having a net negative charge, it can interact with the Zn^{2+}-Phos-tag complex that may explain the appearance of slower bands in the P13 fraction from the S134D mutant. The fact that we did not identify a phosphorylated peptide by mass spectrometry, it is not a contradictory result. It is well known that this technique, like many others, is not fool proof. There is evidence that close to 50% of the predicted peptides are not identified by mass spectrometry, a result that it is even lower for the identification of post-translational modifications (Fricker, D. L. 2015. Limitations of mass spectrometry-based peptidomic approaches J Am Soc Mass Spectrom. 26(12): 1981-1991). The strength of our proteomics results is shown by the clear identification of two very similar peptides in the three analysed samples, that

© 2023. Published by The Company of Biologists under the terms of the Creative Commons Attribution License (https://creativecommons.org/licenses/by/4.0/).

correspond to one of the cytoplasmic loops. These results suggest that this region is more likely to be digested by trypsin, generating the identified peptides.

3. The results of synthetic mutation experiments are confusing (Figure 6C). In Sec23-1 erv14∆ strain, reconstitution of Erv14 rescued growth defects. However, opposite results, i.e. further inhibition of growth, were shown in Sec16- 2 erv14∆ and Sec13-1 erv14∆ strains. As Sec23, Sec13, and Sec16 primarily involve in the same pathway, this inconsistence needs to be reconciled.

The results shown on Figure 7C, in general, agree with the results reported by Powers and Barlowe (2002). The inhibition generated by Erv14 on the *sec16Δ* mutant shown in Fig. 7C might be due to the different medium that was used in this assay (YNB) due to the conditions required to maintain the plasmid (pAG425-GPD-3HA) that was used to clone Erv14, Erv14-S134A and Erv14-S134D. The lack of Leu in this medium might establish certain conditions that lead to this response. As we show in the following figure, when the cells were grown in YNB medium at 23°C, the *sec16Δ* mutant (with Erv14 intact) grew less than the double sec16Δerv14Δ mutant, response that was reversed at higher temperatures (yellow rectangle), and similar to the results reported by Powers and Barlowe (2002). A similar response can be given to the results we obtained with the double *sec13Δerv14Δ* mutant (Fig. 7C). Independently of these, apparently, controversial results, the important message derived from them is that mutations on S134 exacerbated cell growth, particularly in the *sec16Δ* and *sec23Δ* mutants, confirming the importance of an intact C-terminus in Erv14 for the correct functioning of the COPII system.

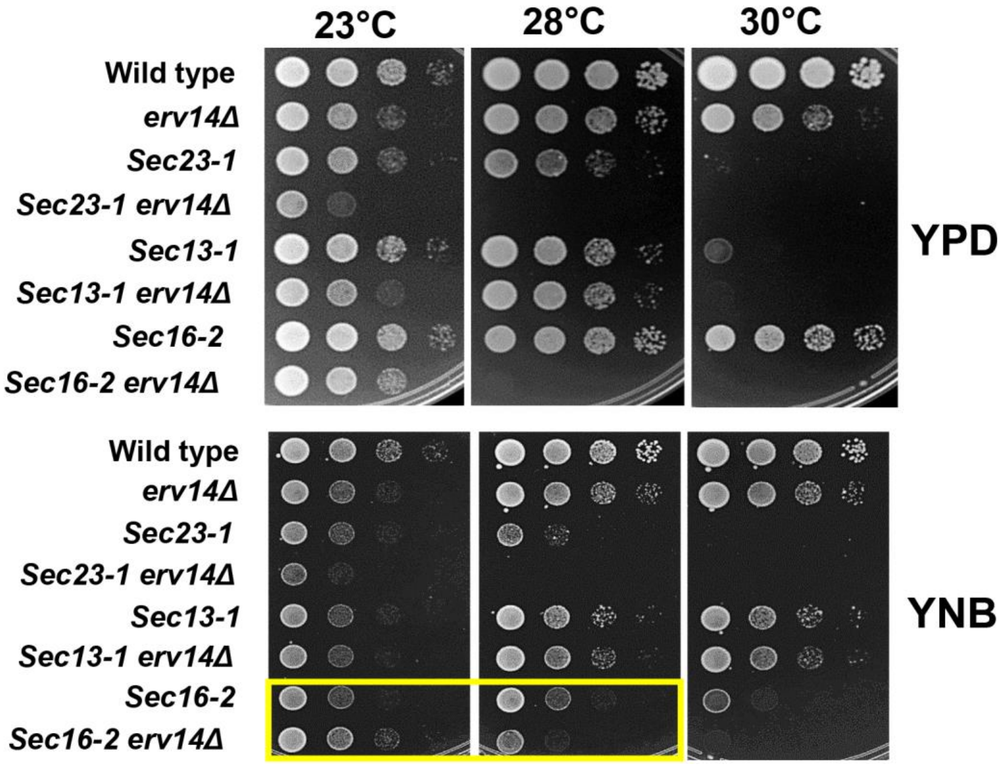

4. Data of ER structural changes caused by Erv14 mutations are weak (figure 5). Only one low-mag EM image was shown in each condition. High-mag images and quantification of these changes are required to support the conclusion. In addition, it would be nice to see the secretory compartments in these conditions using EM, which will be synergistic to vesicle formation results (Figure 6A).

Answering the comment from the Reviewer, we did observe the modifications in the structure of the ER shown, in now Fig. 6, in more than one cell type, as it is now shown in Fig. S5 and the data shown in the Table, where we quantified the modifications on the ER structure in a larger number of cells, results that confirm the altered ER structure in the

© 2023. Published by The Company of Biologists under the terms of the Creative Commons Attribution License (https://creativecommons.org/licenses/by/4.0/).

S134D and S134A mutants, in comparison to the WT cells. As these alterations in the morphology of the ER are easily observed at low magnification, we do not think it is necessary to acquire images at higher magnification. The Reviewer's comment that it would be nice to have images of the secretory system showing morphological changes that could be associated to the modifications generated by the mutations, however, this task is not easy to obtain and would require much more additional work to obtain the required images where the COPII vesicles could be observed. However, with the quantification of the number of cells that showed a modified ER in the two S134 mutants compared to the WT cells we believe our data are solid and support our results

---

Second decision letter

MS ID#: JOCES/2022/260527

MS TITLE: The C-terminus of the cargo receptor Erv14p affects COPII vesicle formation and cargo delivery

AUTHORS: Daniel Gomez-Lagunas, Carolina Yanez-Dominguez, Guadalupe Zavala-Padilla, Charles K Barlowe, and Omar Pantoja
ARTICLE TYPE: Research Article

I am happy to tell you that your manuscript has been accepted for publication in Journal of Cell Science, pending standard ethics checks.

© 2023. Published by The Company of Biologists under the terms of the Creative Commons Attribution License (https://creativecommons.org/licenses/by/4.0/).
